# *Ixora* (Rubiaceae) on the Philippines - crossroad or cradle?

**DOI:** 10.1186/s12862-017-0974-3

**Published:** 2017-06-07

**Authors:** Cecilia I. Banag, Arnaud Mouly, Grecebio Jonathan D. Alejandro, Birgitta Bremer, Ulrich Meve, Guido W. Grimm, Sigrid Liede-Schumann

**Affiliations:** 1 Department of Biological Sciences, College of Science, University of Santo Tomas, España Boulevard, 1015 Manila, Philippines; 2 Research Center for the Natural and Applied Sciences, University of Santo Tomas, España Boulevard, 1015 Manila, Philippines; 3 Bergius Botanic Garden, Stockholm University, 106 91 Stockholm, Sweden; 4 Department of Plant Systematics, University of Bayreuth, Universitätstraße 30, 95440 Bayreuth, Germany; 5 UMR CNRS 6249 Chrono-environnement, Université Bourgogne - Franche-Comté, 16 Route de Gray, 25030 Besançon cedex, France; 6 Orléans, France

**Keywords:** Huxley’s line, incongruent genealogies, island biogeography, *Ixora*, molecular systematics, incomplete lineage sorting, Philippines, phylogeny, Rubiaceae, Wallace’s line

## Abstract

**Background:**

The Philippine archipelago is globally one of the most important model island systems for studying evolutionary processes. However, most plant species on this archipelago have not yet been studied in sufficient detail. The main aim of this study is to unravel the evolutionary history and biogeographic relationships of the Philippine members of the pantropical genus *Ixora*.

**Results:**

The complex plastid and nuclear divergence patterns in Philippine *Ixora*, documented using tree and network approaches, reveal a highly dynamic evolution in *Ixora*, involving several phases of radiation and recolonization. Philippine *Ixora* comprises at least five lineages, of which one is most closely related to species from Wallacea, and the remaining four to species from Asia.

**Conclusions:**

Our study highlights the importance of Philippine species for understanding phytogeographic patterns in the Indomalayan-Australasian eco-region. The overall genetic differentiation, as well as the incongruence between genealogies based on the biparentally inherited nucleome and the maternally inherited plastome in *Ixora*, reflect the complex tectonic history of the Philippine archipelago. The *Ixora* lineage related to Wallacean species supports the delimitation of different ecozones along Huxley’s line, because it is absent from Palawan. The remaining four lineages are all allied with Asian taxa, reflecting several waves of colonization. Close relationships between some widespread Philippine species and locally adapted narrow endemics suggest that the widespread, genetically diverse species act as pools for the formation of new species in a process of ongoing speciation. Our results suggest that the species concepts of some of the more widespread taxa need to be revised.

**Electronic supplementary material:**

The online version of this article (doi:10.1186/s12862-017-0974-3) contains supplementary material, which is available to authorized users.

## Background

The importance of the Philippine archipelago for Southeast Asian biogeography was first recognized by Alfred Russel Wallace when he distinguished the Australian and Indian regions [1, 2]. He placed the Philippines in the Indian region, but considered them “in some respects of doubtful location”. Later, he moved the Philippines into the Oriental region [3].

With almost 6000 endemic plant and more than 500 endemic vertebrate species, the Philippines constitutes one of the 25 biodiversity hotspots for conservation priority and is among the leading ten hotspots regarding the number of endemics [4]. However, the complex geological history of the more than 7000 islands stretching over nearly 2000 km in North-South direction has made it difficult to understand the diversification of the rich plant and animal biodiversity in the country [5]. Nevertheless, biogeographers, population geneticists, conservation biologists, and phylogeneticists have been intrigued by the archipelago and its diverse endemic species, and have used it as a model system for addressing a variety of conceptual questions relating to evolutionary history. Consequently, the Philippine archipelago has become one of the globally important model island archipelagos for studying evolutionary processes of diversification [6]. Studies utilizing robust and well-sampled phylogenetic analyses as a basis for understanding the complex biogeographical histories of Philippine plants and animals have advanced in the last two decades; however, most of them are dedicated to animals [7–10]. Biogeographical studies including Philippine plants were conducted only for a few genera such as *Cyrtandra* J.R.Forst. & G.Forst. (Gesneriaceae [11]), *Rhododendron* L. (Ericaceae [12]), *Begonia* L. (Begoniaceae [13, 14]), and *Aglaia* Lour. (Meliaceae [15]).

In the Philippines, Rubiaceae is the most diverse family, and 443 (83%) of the 535 species found in the country are endemics [16]. Species diversity, phylogenetic and biogeographical relationships of Philippine Rubiaceae have received renewed interest in recent years. *Greeniopsis* Merr. (Ixoroideae: Aleisanthieae) has been identified as an endemic Philippine genus with its closest relatives (*Aleisanthia* Ridl. and *Aleisanthiopsis* Tange) in Southeast Asia [17]. Likewise, the endemic Philippine genus *Antherostele* Bremek. (Rubioideae: Urophylleae) is most closely related to a set of Southeast Asian genera (*Maschalocorymbus* Bremek., *Pleiocarpidia* K.Schum., *Praravinia* Korth., *Pravinaria* Bremek., *Urophyllum* Jack ex Wall. [18]). In contrast, the endemic Philippine genus *Villaria* Rolfe (Ixoroideae: Octotropideae) forms a well-supported clade with the Southeast Asian genus *Hypobathrum* Blume and the West African genus *Pouchetia* A.Rich. [19].

None of these endemic genera comprise more than six species, and little is known about species diversity, phylogenetic and biogeographical relationships of the Philippine representatives of other, larger Rubiaceae genera. The pantropical genus *Ixora* L. (Ixoreae [20]) is the third largest genus in the family Rubiaceae, with approximately 530 species [16], most of them shrubs or small trees in the understorey of tropical forests. Approximately 280, (possibly up to 300) species occur in tropical Asia [21], with 44 species in India [22], 38 in Thailand [23] and 66 species on Borneo alone [24]. In contrast, only 37 species are known from continental Africa, about 40 species from Madagascar and 35 species from tropical America [21, 25]. *Ixora* is one of the largest Rubiaceae genera on the Philippines, and one of the best recognizable: Morphologically, it is characterized by a combination of articulate petioles, terminal trichotomously branching inflorescences, narrowly tubular 4-merous flowers, contorted aestivation, a single ovule per locule, and drupaceous fruits and seeds with a large adaxial excavation [21]. However, identification at species level is much more difficult [21]. While several taxonomic treatments of *Ixora* are available for specific geographical regions such as Africa [21], Madagascar [25], the Marquesas Islands [26], and Australia [27], a revision of the continental Asian taxa is lacking. Therefore, species delimitation is not yet fully understood, and the actual number of species is still unknown [28].

Previous phylogenetic studies have clarified the tribal placement and circumscription of the genus [20, 29–31]. Mouly et al. [31] resolved *Ixora* species into two large lineages, an Asian-Pacific lineage (43 species covered) and an Afro-Neotropical lineage (34 species covered). Tosh et al. [32] recently investigated the evolutionary history of Afro-Madagascan *Ixora* and recovered two separate lineages of Madagascan taxa*.* They [32] estimated an Ixoreae crown age of 17 million years ago (Ma) and dated the onset of divergence between the Asian-Pacific clade and the remainder of the genus as 15 Ma, indicating a mid-Miocene origin (cf. [33]) for the lineages, in agreement with the results of Mouly [28]. No samples from the Philippines have been included in any study so far.

In the Philippines, the genus *Ixora* provides an exemplary case, with a particularly high number of endemic species known from the country (30 out of 41 species [34, 35]). The only available prior account was an enumeration by Merrill [36], more than 85 years old and outdated (e.g. [20, 37–39]). Preliminary investigation of type material and available herbarium specimens showed that species of *Ixora* in the Philippines are distinguished based on subtle differences of the inflorescences and morphoclines rather than discrete characters, e.g. length ratio of the corolla tube vs. corolla lobe, and the pubescence of the inflorescence [35]. This corresponds to De Block’s [21] observations regarding their African congeners.

In this study, we include for the first time a wide range of Philippine *Ixora* species in a phylogenetic analysis using sequence data from two chloroplast regions, the *rps*16 intron and the trnT-F region including the *trn*T*-﻿trn*L and *trn*L-*trn*F intergenic spacers and the *trn*L intron, and the 5′ external transcribed spacer (ETS) and internal transcribed spacers (ITS1, ITS2) of the nuclear-encoded 35S rDNA cistron. We interpret our results in the light of the hitherto known patterns of faunal and floral migrations and evolution in the Philippines. We address the following questions. (1) Are the Philippine *Ixora* species monophyletic? (2) Are phylogenies derived from chloroplast and nuclear DNA congruent? (3) Which species or groups of species are the closest relatives of the Philippine *Ixora* species? (4) Does the phylogenetic pattern of Philippine *Ixora* have wider implications on the biogeographic history of the Philippines?

## Results

### Outgroup sampling and ingroup sequence characteristics

One of the non-coding gene regions used here, the nuclear-encoded ITS1, cannot be aligned across all Ixoroideae, and alignment of the most divergent and length-polymorphic plastid region, the *trn*T-*trn*L intergenic spacer, is difficult in some parts. Also, the other two nuclear spacers, the ITS2 and the ETS, include sequence portions that will lead to ambiguous alignments when incorporating all Ixoroideae data. Nonetheless, we could infer four guide trees based on the harvested data (ETS, ITS excluding non-alignable regions, *rps*16 intron, entire trnT-F region), which confirmed that our outgroup selection includes the closest relatives of *Ixora* with best-possible data coverage on the gene regions used here (see Additional file 1).

The 151 new sequences generated in this study were combined with sequences previously generated and used by Mouly et al. [30, 31] and Tosh et al. [32] resulting in a total of 264 sequences of 78 *Ixora* samples, representing approximately 60 *Ixora* species. Levels of genetic variation between species were generally low for all investigated regions. The total number of parsimony informative characters (PICs) ranged from 40 in *rps*16 to 122 in ETS for the ingroup; the number of distinct alignment patterns (DAPs) ranged from 209 in *rps16* to 450 in trnT-F. In the concatenated cpDNA dataset, 265 characters (10%) were variable, with 360 characters (32.4%) variable in the nuclear dataset. The characteristics of the individual chloroplast and nuclear regions are listed in Table 1.

**Table 1:** Information for phylogenetic analyses (*rps*16, trnT-F, ITS and ETS, ingroup only)

	*rps*16	trnT-F	ITS	ETS
No. of sequences investigated	74	75	73	65
No. of new sequences	39	40	40	32
Range of sequence length (bp)	593–876	767–1744	552–791	282–436
Length of aligned matrices (bp)	847	1798	676	436
Proportion of gaps and undetermined characters	14.4%	16.3%	6.3%	5.5%
Number of variable characters	96 (11.3%)	169 (9.4%)	175 (25.9%)	185 42.4%)
Number of PIC	40 (4.7%)	70 (3.9%)	106 (15.7%)	122 (28.0%)
Number of DAP	209	450	248	260

*Abbreviations*: *DAP* distinct alignment patterns, *PIC* parsimony-informative characters

### Incongruence and congruence between nuclear and plastid genealogies

**Fig. 1:**
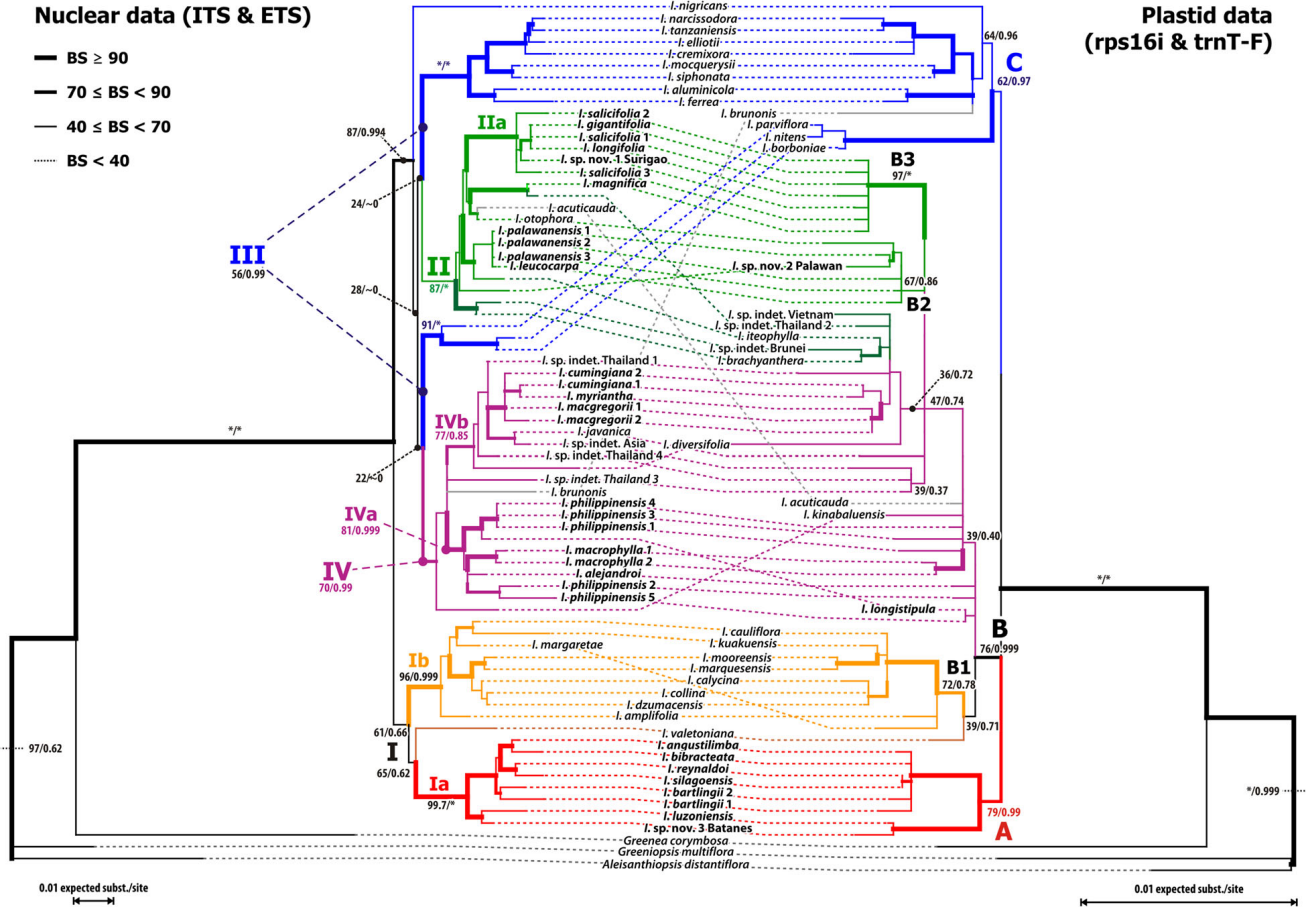
Tanglegram showing maximum likelihood trees based on the nuclear (*left*) and the plastid (*right*) datasets, after exclusion of the cultivated species. Branch numbers indicate bootstrap support values and posterior probabilities for the main lineages. Major clades are labelled with *roman numbers* for nuclear data, and *uppercase letters* for the plastid data (subclades use *lowercase letters* and *Arabic numbers*). Colours highlight main lineages characterized by coherent combinations of nuclear and plastid genotypes: *red*, lineage endemic to the Philippines (with nucleome type I and plastome type A); *orange*, putative South Asian-Australasian sister lineage of the *red* lineage (I/B); *purple*, Southeast Asian lineage with Philippine members (II/B), *blue*, African-New World lineage (III/C); *green*, East Asian lineage with members on Palawan and in the Philippines (IV/B). Same colouring scheme is used in subsequent figures and tables

**Fig. 2:**
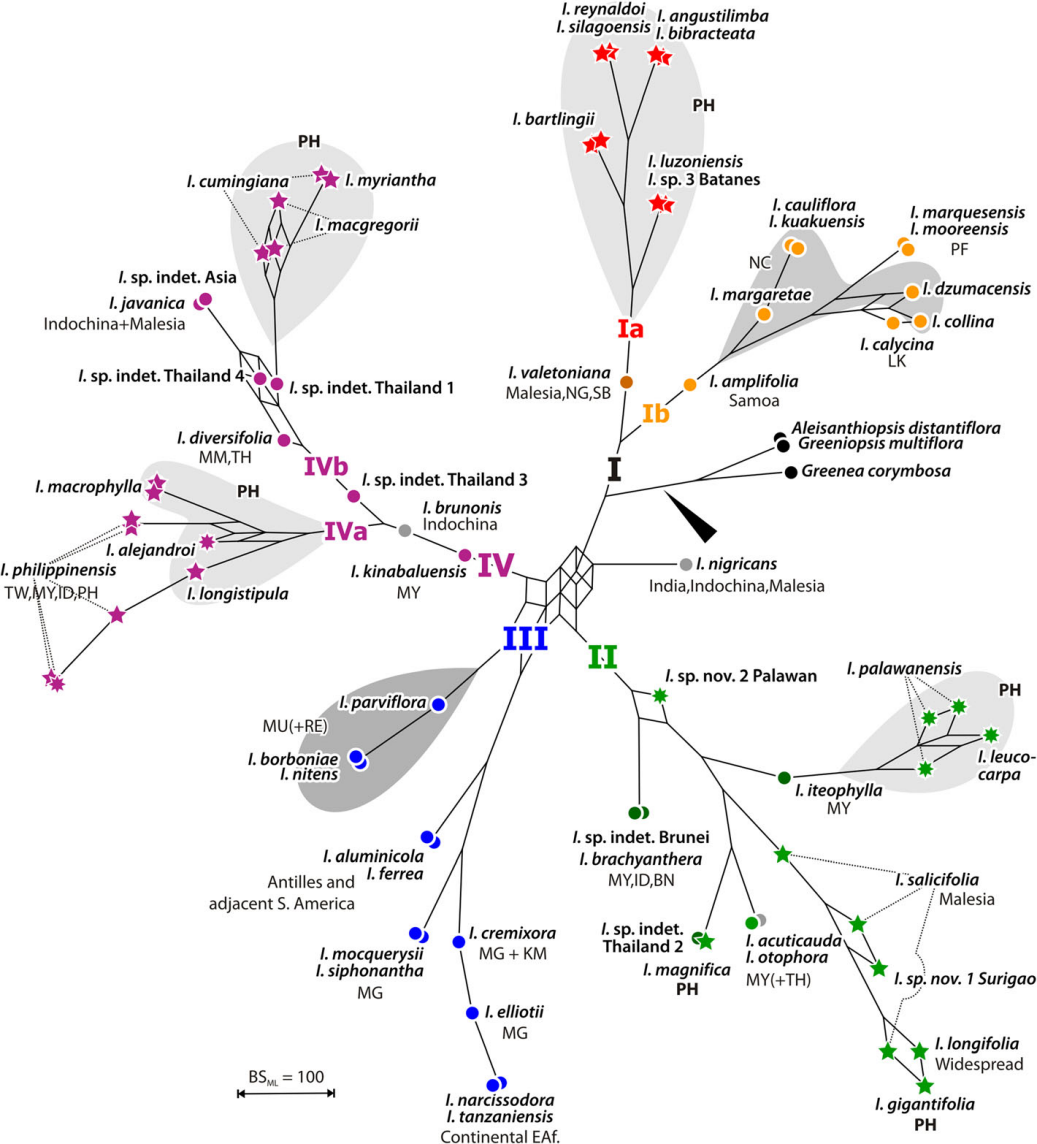
Bootstrap support network, nuclear data. Ambiguous signal in the nuclear data (excluding cultivars) visualized using bipartition (bootstrap support) networks [80, 82], a special from of consensus networks in which the edge lengths are proportional to the frequency of the corresponding phylogenetic split in the bootstrap (BS) replicate sample The bootstrap support network is based on 400 BS replicate trees inferred from the nuclear data.. Members of major lineages (see Fig. 1) coloured accordingly, outgroups in *black*. *Circles*, non-Philippine individuals; *7-pointed stars*, Palawan samples; *5-pointed stars*, other Philippine samples. (Corresponding posterior probability networks can be found in Additional file 2)

**Fig. 3:**
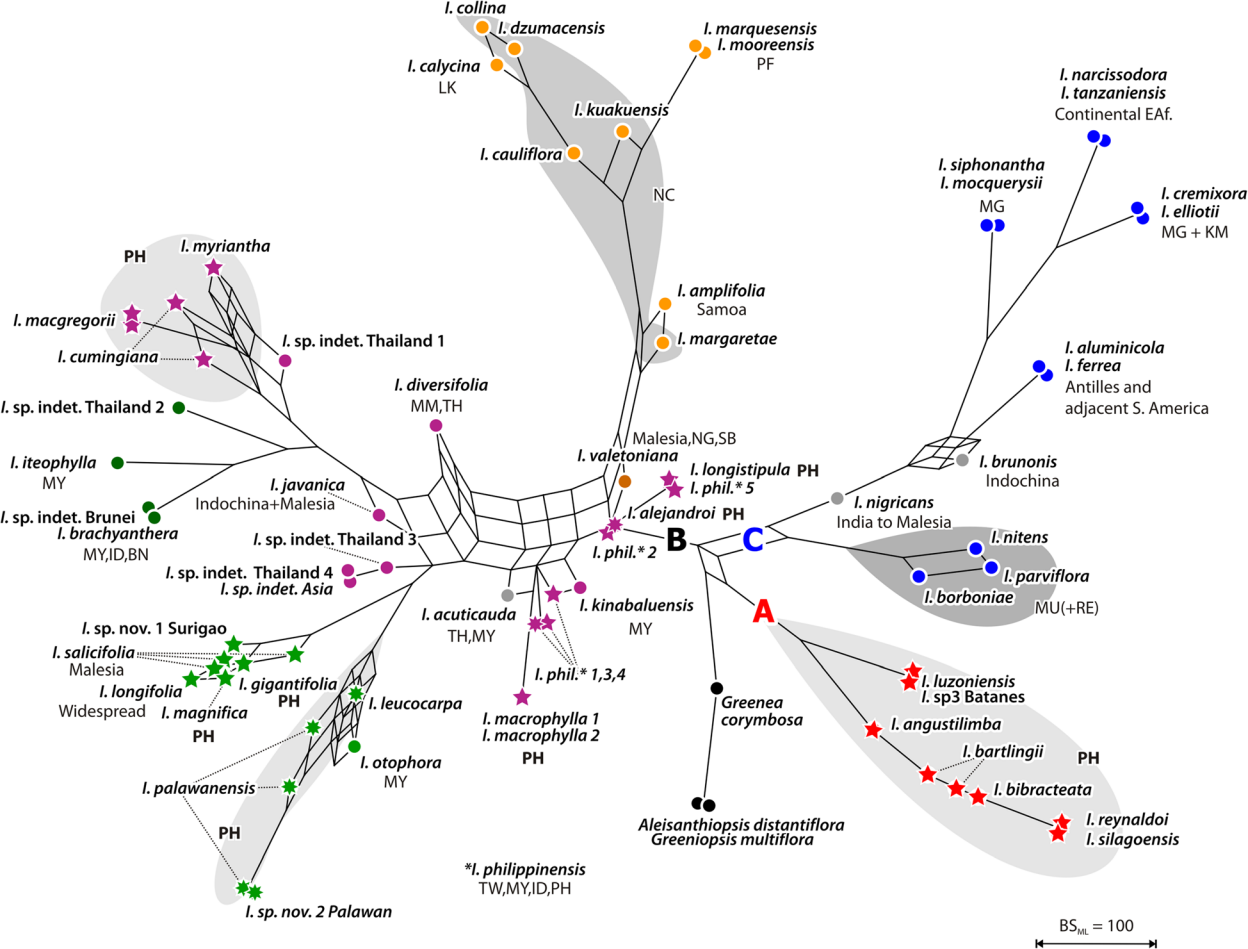
Bootstrap support network, plastid data. Ambiguous signal in the plastid data (excluding cultivars) visualized using bipartition (bootstrap support) networks, a special from of consensus networks in which the edge lengths are proportional to the frequency of the corresponding phylogenetic split in the bootstrap (BS) replicate sample. The bootstrap support (bipartition) network is based on 700 BS replicate trees inferred from the plastid data. Members of major lineages (see Fig. 1) coloured accordingly, outgroups in *black*. *Circles*, non-Philippine individuals; *7-pointed stars*, Palawan samples; *5-pointed stars*, other Philippine samples. (Corresponding posterior probability networks can be found in Additional file 2)

Phylogenetic analyses of individual gene regions generate largely unresolved and poorly supported phylogenetic trees (data not shown). Concatenation of the nuclear data and the plastid gene regions respectively leads to better resolved phylogenetic trees (Fig. 1) and limits the number of scenarios of potential phylogenetic relationships between species and clades (Figs. 2 and 3).

Two large, mutually monophyletic lineages, an Asian-Pacific clade (I) and an Asian-Afro-Neotropical clade (clades II–IV), are inferred based on the nuclear dataset (Figs. 1a and 2). Based on the plastid data, three main clades emerge: a Philippine clade (A), a mixed Asian-Pacific clade (B), and a clade including the African and New World species (clade C; Figs. 1b and 3). The nuclear tree suggests a split between the Indian-Mascarene species and the remainder of the African-New World clade. A nuclear clade III corresponds to the plastid clade C. This is the best-supported (via bootstrapping, BS) and most probable (regarding Bayesian inferred posterior probabilities, PP) clade (Figs. 1 and 2). In both phylogenies, the American taxa *I. aluminicola* and *I. ferrea* are nested in the African subtree(s). Taking the nuclear and plastid inferences together, four or five main lineages can be defined based on the exclusive combination of nuclear and plastid signatures: Ia/A (red in Figs. 1, 2 and 3), Ib/B1 (orange), II+IV/B (green and purple), and III/C (blue). The nuclear clade Ia corresponds exactly to the plastid clade A. Based on nuclear data, this clade (Ia) is sister to a clade of Asian-Pacific species (Ib) with B-type plastids (clade B1 in Fig. 1). Based on plastid data, this clade (A) is sister to the remaining taxa, reflecting a deep incongruence. Although different in their nuclear signatures, the members of the nuclear clades II and IV share the same plastid. Consistently, the plastid haplotypes of members of clade II appear to be generally more derived (to different extents) and include unique ribo−/haplotype combinations (II/B2 and II/B3; see below) as reflected by their placement in the plastid tree and the support of critical branches (Figs. 1, 2 and 3).

Both the nuclear and chloroplast maximum likelihood (ML) trees (Fig. 1a and b; see Additional file 2 for the corresponding trees including cultivars) placed the 35 samples (24 species) of Philippine *Ixora* in five distinct subtrees. The positions and support of the five corresponding clades, however, differ between the two datasets. Lineage Ia/A is exclusively Philippine. The remaining four clades comprising Philippine species mixed with Southeast Asian species are part of lineage(s) II+IV/B. The structure of the nuclear clade II reflects two radiations with Philippine species. While the exclusively Philippine subclade IIa matches exactly the chloroplast clade B3, composition and arrangement of the *I. palawanensis* subclade (clade B2) differs between the two datasets. Using the nuclear data the Malayan *I. iteophylla* is included in this clade but *I. otophora* and *I.* sp. nov. 2 “Palawan” are excluded. A similar situation is found in nuclear clade IV; one subclade (IVa) is exclusively Philippine, while the other (subclade IVb) is comprised of both Philippine and several Southeast Asian species. In the chloroplast tree, these two radiations do not correspond, and the involved species are largely unresolved.

Multiple accessions of the same species are placed in the same subclades (*I. palawanensis*, *I. salicifolia, I. macgregorii, I. cumingiana*); but only *I. bartlingii* and *I. macrophylla* are resolved as actual sisters, irrespective of the data used (nuclear or plastid). The highest variability is encountered in the five accessions of *I. philippinensis* which fall into two groups with (to some extent) different plastid and nuclear signatures.

### Signal ambiguity in nuclear and plastid data sets

Detailed inspection of split patterns in the bootstrap samples (Figs. 2 and 3; datasets excluding cultivars) demonstrate that the nuclear types are generally more distinct than the plastid types of the same specimens; this is illustrated in the more tree-like general structure of the nuclear-based bootstrap network. Signal ambiguity in the nuclear data relates to the initial radiation within the core group *Ixora* and the initial diversification of clade III. The plastid signal is not sufficiently clear either to resolve several backbone relationships. Most importantly, the signal from many Philippine taxa of clade B commonly is ambiguous. Other taxa (or pairs of taxa) inflicting topological ambiguity at deeper nodes in the *Ixora* core group plastome are *I. diversifolia, I. javanica, I. acuticauda, I. kinabaluensis,* and *I. valetoniana.*

Individual taxa of unclear affinity are: 1) *I. nigricans* from India, with somewhat inconclusive nuclear signals but an African-American -type chloroplast; 2) *I. brunonis* from Thailand with Southeast Asian nuclear signals but an African-American type chloroplast; and 3) *I. acuticauda* from Borneo with incongruent Asian nuclear and chloroplast types.

Further topological ambiguity arises from to the cultivated taxa which occupy markedly different positions in the nuclear- and plastid-based inferences (Additional file 2). The cultivars *Ixora finlaysoniana*, *I. pavetta*, *I. casei,* and *I. chinensis* are nested in the Asian (including Philippines) nuclear-based clade IV; but their plastid affinity lies with the New-World African clade C. Similarly, *I. brunonis* from Thailand is the poorly supported sister of *I. casei* and *I. chinensis* in the nuclear tree (all members of clade IV), while it is the well-supported sister of *I. finlaysoniana* in the cpDNA tree (subclade of clade C). The accession of *I. finlaysoniana* cultivated in the Philippines always formed a strongly supported clade with an accession cultivated in Tanzania. The last cultivar, *Ixora coccinea*, retains its association with a second clade of Asian (including Philippine) species in both trees (clades II, clade B), but changes position inside this clade.

### Biogeographic patterns in *Ixora* with special reference to Philippine taxa

**Fig. 4:**
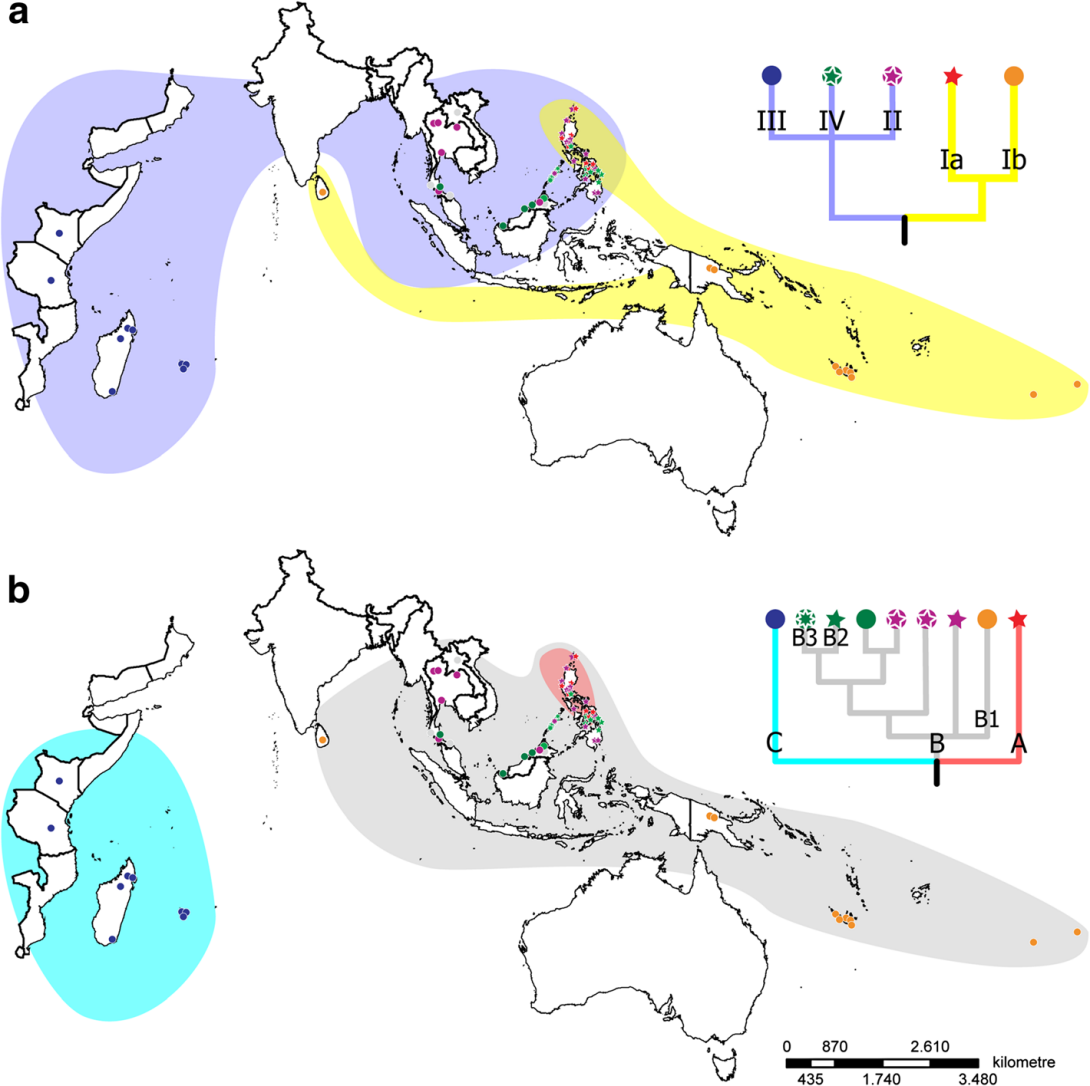
Overview map including all analysed samples. Geographic distribution of nuclear-inferred (**a**) and plastid-inferred (**b**) lineages within sampled Ixora. Phylogenetic relationships of distinguished nuclear and plastid lineages are depicted as schematic cladograms, root as defined by outgroups (branches with non-high support collapsed to polytomies). Members of major lineages (see Fig. 1) coloured accordingly, *grey*: not genotyped individuals. *Circles*, non-Philippine individuals; *7-pointed stars*, Palawan samples; *5-pointed stars*, other Philippine samples

**Fig. 5:**
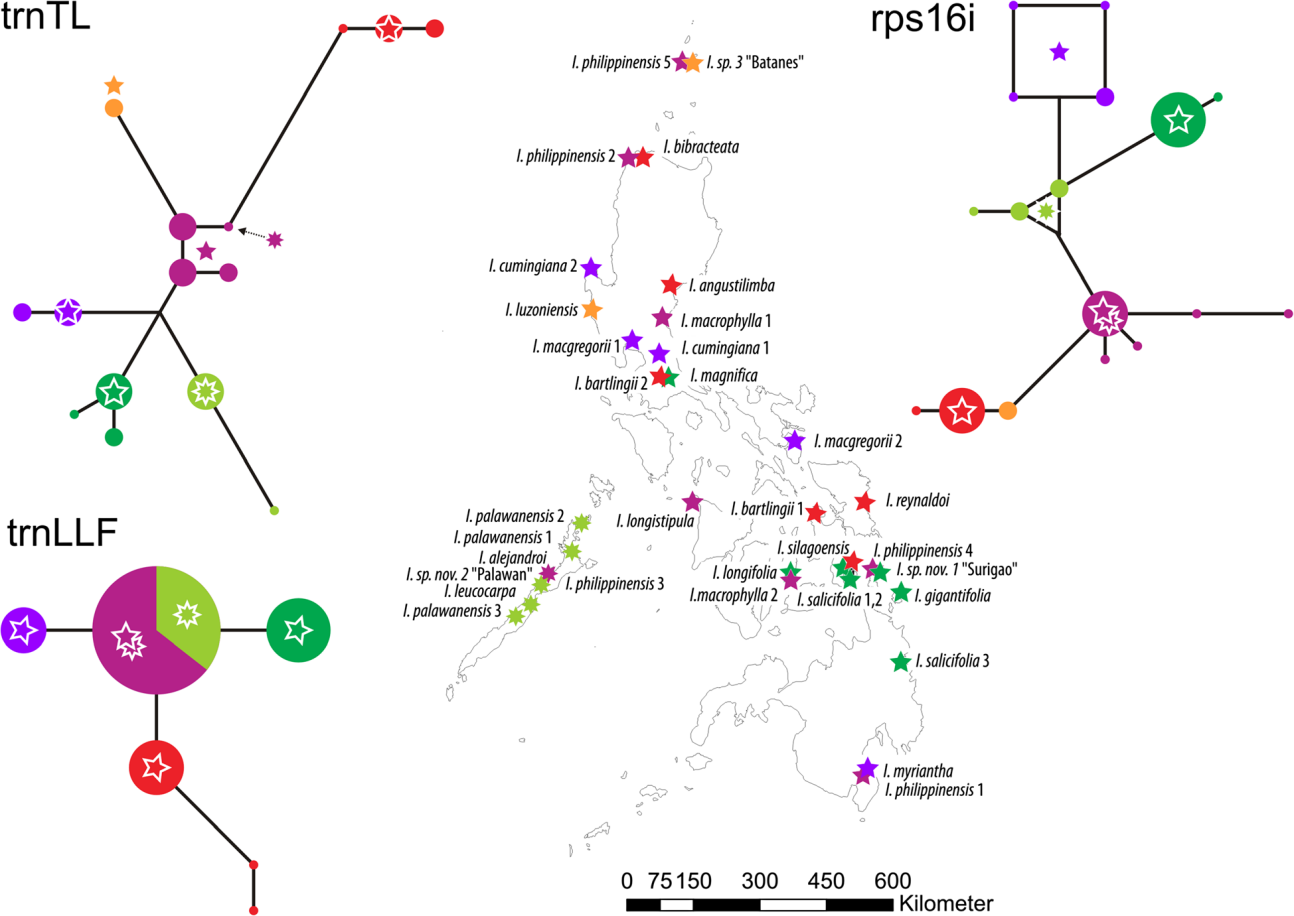
Close-up on Philippine archipelago. Geographic distribution of main *Ixora* lineages (coloured accordingly, see Figs. 1, 2 and 3) on the Philippines (**a**) and hypothetical evolutionary pathways of plastid haplotypes as inferred using median networks of individual gene regions. **b** *trn*T-*trn*L intergenic spacer. **c** trnLLF region including *trn*L intron and *trn*L-*trn*F intergenic spacer. **d** *rps*16 intron. *Circles*, non-Philippine individuals; *7-pointed stars*, Palawan samples; *5-pointed stars*, other Philippine samples

In the Philippines, distantly related nuclear lineages occur sympatrically (Fig. 4a, stars), indicating that natural populations of the main lineages are genetically isolated. Plastids in *Ixora* are geographically sorted. With a few exceptions, each nuclear lineage (Ia, Ib, II, III, and IV) carries one sort of plastid haplotype (A, B or C) (Figs. 4 and 5). The outgroup-inferred root suggests an initial split in *Ixora* between a lineage that today occurs from the Pacific Islands to the Philippines (except for Palawan) with an outlier in Sri Lanka (yellow in Fig. 4a), and the rest of the genus (light blue). Plastid variation indicates substantial genetic drift between African (lineage III/C; blue signatures in Fig. 4b) and South Asian-Indomalayan members (green and purple signatures in Fig. 4b) of the genus in general, and the two Pacific-Philippine potential sister lineages Ia/A and Ib/B1 (red and orange signatures, respectively). Plastid signatures indicate that the now mostly Pacific lineage Ib/B1 (orange) and the South Asian-Indomalayan group (lineage[s] II + IV/B; green, purple) within *Ixora* evolved within or near to the same geographically restricted area(s) (grey plastid clade, Fig. 4b). At the time of this divergence, both the African (blue) and the exclusively Philippine (red) *Ixora* were geographically isolated from the main bulk of South Asian-Indomalayan *Ixora* (Fig. 4b).

## Discussion

### Relationships of the Philippine *Ixora* species

Well-supported incongruence between the nuclear and the plastid datasets prevents the analysis of a combined dataset ([40, 41], and references therein). Therefore, the two different genealogies were explored separately.

Our main nuclear clades, the Asian-Pacific clade (I) and the Asian-Afro-Neotropical clade (II–IV), correspond to those of Mouly et al. [31], who also found an Afro-Neotropical and a Pacific clade. However, while Mouly et al. [31] inferred a poorly supported monophyletic Asian clade, our greatly increased sampling of Asian, and in particular Philippine taxa, shows that Asian (including Philippine) species occur in both major clades in *Ixora*.

The species from the Philippines are represented in five different lineages, corresponding to three major ribo−/haplotypes in the nuclear and the chloroplast dataset. Two of these ribo−/haplotypes are shared with mainland Southeast Asian species, while the third one is a rather distant relative (putative sister lineage) of an Asian-Pacific lineage. Thus, the phytogeographical relationships of the *Ixora* species found in the Philippines are fully decoupled.

**Fig. 6:**
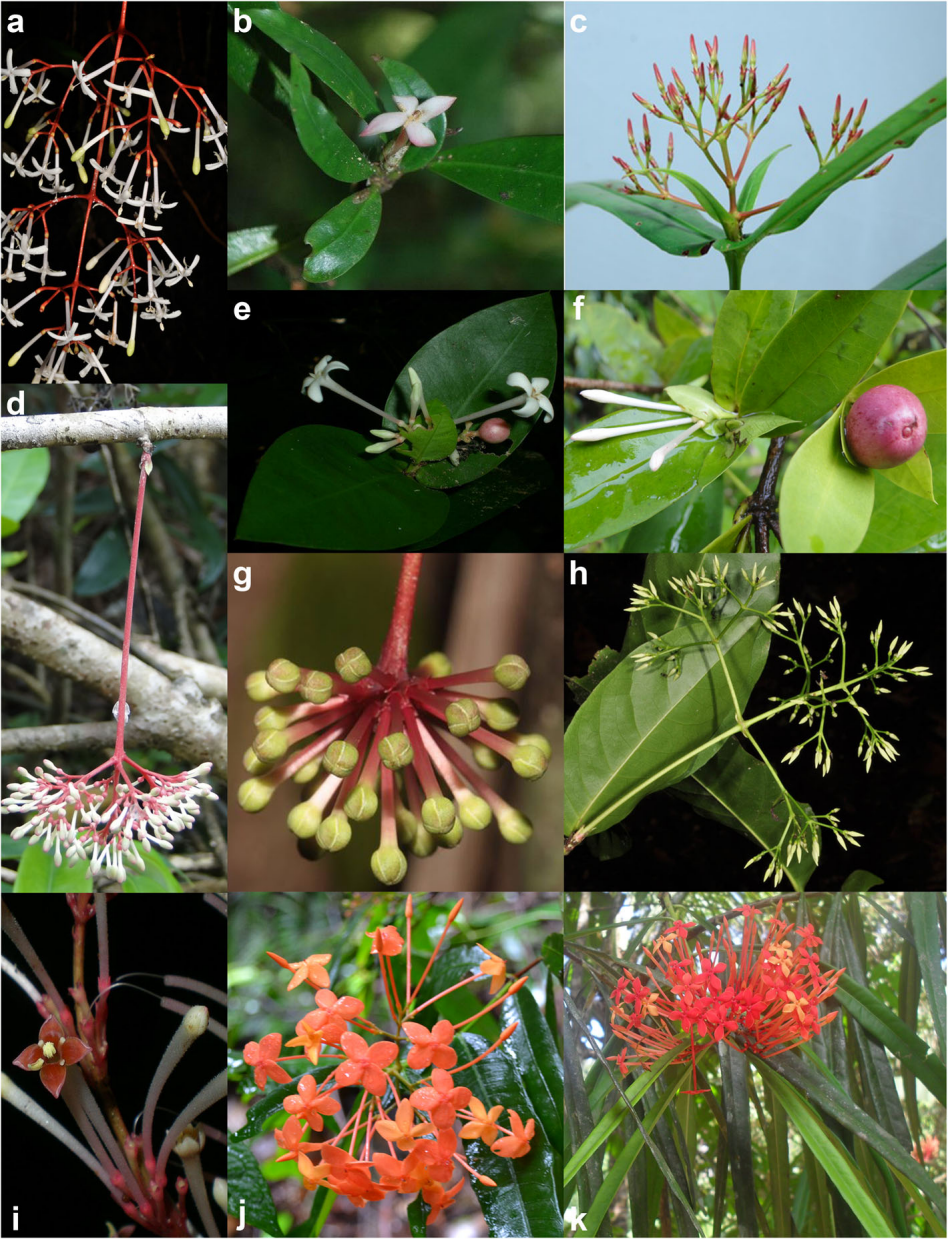
Examples of species of the five subclades of the Philippine *Ixora*. **a** *Ixora bartlingii* Elmer (Pelser et al., 2011 [92]); **b** *Ixora angustilimba* Merr. (Banag 11-032, USTH); **c** *Ixora silagoensis* Manalastas, Banag & Alejandro (Banag 12-037, USTH); **d** *Ixora macrophylla* Bartl. ex DC. (Banag 11-053, USTH); **e** *Ixora philippinensis* Merr. (Tandang DT548, PNH); **f** *Ixora philippinensis* Merr. (Alejandro 11-102, USTH); **g** *Ixora longistipula* Merr. (Pelser et al., 2011 [92]); **h** *Ixora cumingiana* Vid. (Pelser et al., 2011); **i** *Ixora alejandroi* Banag & Tandang (Tandang MH1707, PNH); **j** *Ixora palawanenis* Merr. (Medecillo MPM 471, USTH); **k** *Ixora salicifolia* DC. (Banag SU002, USTH). — Credits: P. Pelser (A, H); G. Alejandro (B, D, F); J. Dela Bajan (C); D. Tandang (E, I); R. Bustamante (G); C. Banag (K)

Lineage Ia/A comprises a strongly supported, genetically isolated group of endemic Philippine *Ixora* species that possibly share a common, potentially widespread (see plastid signatures) ancestor with a lineage (Ib/B1) composed of species from the wider Pacific area, which also includes *I. calycina* from Sri Lanka and southern India. *Ixora angustilimba* and *I. bibracteata* are strongly supported sister species, both characterized by solitary (Fig. 6b) or at the most, three, flowers in an inflorescence supported by bracts [42, 43]. Sister to these two species are two recently described species, *I. reynaldoi* and *I. silagoensis* [44, 45]. Both species are characterized by subsessile leaves, sessile or shortly pedunculate, erect and lax inflorescences (Fig. 6c). Nested in this group are the two samples of *I. bartlingii* characterized by long-pedunculate, pendulous inflorescences bearing numerous flowers (Fig. 6a). The sister clade to this group contains *I. luzonensis* and *I.* sp. indet. 3 “Batanes”, which differ from their relatives by their shortly pedunculate and lax inflorescences.

The nuclear clade II, one of the two lineages with exclusively B-type plastids, contains two well-supported subclades, one of them exclusively consisting of Philippine (without Palawan) species (subclade IIa) with derived plastids (plastid clade B3; Figs. 1, 3 and 5). The ‘plesiomorphic’ plastids found in other members of the same lineage including all samples from Palawan, cause the ambiguous, but weak signals along the proximal part of the B-type subtree, characterizing, to various degrees, all members of clades II and IV (Figs. 1 and 3). The Palawan clade B2 (subclade of clade II in the nuclear tree) comprises *I. palawanensis* and *I. leucocarpa* (Figs. 1, 2 and 3). *Ixora* sp. nov. 2 “Palawan” is sister to these species included in clade B2, but outside the *I. palawanensis*–*I. leucocarpa* subclade in the nuclear tree, where it is sister to the Malayan *I. iteophylla*. Though sharing similar habitats, forested ravines or humid forest, the species of the Palawan clade differ in their flower colour (*I. palawanensis,* salmon-red (Fig. 5j); *I. leucocarpa,* white; *Ixora* sp. nov. 2 “Palawan”, orange).

The three accessions of *I. salicifolia* are all part of clade B3 in the plastid tree and of IIa in the nuclear tree, but are not resolved as discrete clades (Fig. 1). *Ixora longifolia* and *I. gigantifolia* group with *I. salicifolia* 1 in the nuclear tree*,* while *I. salicifolia* 2 and *I. salicifolia* 3 remain unresolved. All species in this group possess derived, similar or identical, haplotypes of lineage B (Fig. 5, Additional file 3) and share morphological traits: pedunculate, erect and compact inflorescences and long-pedicellate flowers (Fig. 6k). *Ixora magnifica*, another Philippine endemic with showy bright red flowers, is resolved with high support as sister to an unidentified accession from Thailand in the nuclear tree, while sharing the characteristic B3 haplotype.

Within the third *Ixora* lineage (IV/B) found in the Philippine archipelago, the differentiation patterns are less clear. Of the widespread species *I. philippinensis,* two samples collected in the northern part of the Philippines (*I. philippinensis* 2 and 5 from Ilocos Norte and Batanes) are sister of the widespread, but monophyletic *I. macrophylla* (Aurora and Cebu) and *I. alejandroi* (Palawan), and are separated from the three samples collected in the southern part of the country (Palawan, Surigao and Davao). In the nuclear tree, the latter are contained within the sister clade of *I. longistipula* (Panay)*,* a species characterized by pendulous inflorescences in which the flowers form a simple, dense head (Fig. 6g). Tosh et al. [32] observed a similar pattern for *I. mangabensis*, in which the populations from northern and southern Madagascar did not form a natural group and exhibited small morphological differences. In *I. philippinensis*, the material collected from the north has longer peduncles and many flowers per inflorescence (Fig. 6e) compared to the (sub-)sessile to shortly pedunculate inflorescences with at most ten flowers per cyme in materials collected from the south (Fig. 6f). Re-evaluation of the taxonomy of *I. philippinensis* is required in view of the genetic differentiation and morphological variation observed between these populations currently united in this species. The Palawan endemic *I. alejandroi* did not group with other species of Palawan (II/B2; Fig. 1). *Ixora alejandroi* (Fig. 6i) is characterized by an elongated cyme with congested secondary axes, reddish brown corolla and stigmatic lobes shortly cleft in the middle; characters which are not known from any other Philippine species [45]. Except for a pubescent inflorescence and non-articulate branching of the inflorescence axes, *I. alejandroi* shares no morphological characters with *I. macrophylla* and *I. philippinensis.* Its plastids are plesiomorphic within the IV/B lineage (cf. Fig. 3, Additional file 3, and median-joining networks in the online supplementary archive).

Subclade IVb (Fig. 1) comprises the two samples of *I. cumingiana* from Luzon, *I. myriantha* (Davao) and two sterile accessions attributed to *I. macgregorii* (Sorsogon)*.* These species share white flowers and lax inflorescences (Fig. 6h), which become more compressed towards the south.

In our study, some species thought to be closely related based on morphology were placed in different subtrees (clades). This is the case with *I. macrophylla, I. bartlingii*, and *I. longistipula,* which are often misidentified in herbarium collections due to their long-pedunculate, pendulous inflorescences with white corollas and overlapping shape of the leaves. However, other morphological characters support their separation in the phylogenetic tree, particularly the articulate, terminal inflorescences of *I. bartlingii* and *I. longistipula* as opposed to the non-articulate, cauli- or ramiflorous inflorescences of *I. macrophylla* (Fig. 6d) as well as the sessile to capitate flowers of *I. longistipula* (Fig. 6g) as opposed to the pedicellate flowers of *I. bartlingii* and *I. macrophylla* (Fig. 6a and d)*.*

It is interesting to note that the most widespread and variable Philippine species (e.g., *I. philippinensis*, *I. salicifolia*) are found in different groups, associated genetically with morphologically related species of narrower distribution. This suggests that these widespread, genetically diverse species might act as pools for the diversification of locally adapted new species in a process of ongoing speciation, as recently shown for *I. margaretae* in New Caledonia [46].

While our investigation provides unequivocal evidence for the polyphyly of the Philippine *Ixora*, the relationships of several species to related species from the Asian mainland remain to be studied in more detail, because the plastid tree (in particular) is not yet well resolved inside clade B. Lineage sorting in this complex group appears yet to be incomplete (Fig. 1). Moreover, our present results indicate that several species from Asia display unusual combinations of nuclear and plastid ribo−/haplotypes (*I. acuticauda* and *I. brunonis* from Thailand, *I. nigricans* from India; Figs. 1 and 2, Additional file 3) that could be indicative of recent or ancient reticulation between major lineages (introgression, hybridization, as already discussed in Mouly et al. [31]). However, an enlarged sampling of Malesian and Indian taxa and an increased number of loci will most likely refine our results for clade B. Ultimately, the combination of additional, more variable plastid markers and the nuclear markers used here should be able to discriminate further between reticulation and incomplete lineage sorting; the latter appears to be a minor issue in *Ixora*, but it may account for the pattern seen in Southeast Asian/Malesian species of lineage(s) II+IV/B. Reticulation, possibly caused by hybridization [31, 32, 47], is indicated in particular for the cultivated species in our nr- and cpDNA trees (Additional file 2) by their conflicting, relatively terminal positions.

### Endemism

Twenty-one of the 24 Philippine species included in this phylogenetic study are endemic to the Philippines. The three non-endemic species are *I. longifolia*, *I. philippinensis,* and *I. salicifolia*. While lineages Ia/A and II/B2 comprise only species endemic to the Philippines, lineages IVa/B, IVb/B and II/B3 contain both species endemic to the Philippines and species also reported from a wider Asian range. In clade IVa, both *I. macrophylla* and *I. philippinensis* are reported from all major Philippine islands including Palawan, and *I. philippinensis* is supposed to also occur in other areas of Malesia, and as far as Taiwan. However, in our analysis, the multiple accessions of *I. philippinensis* occur in two relatively distinct, well-supported nuclear subclades (Fig. 1, Additional file 2). This raises the question whether *I. philippinensis* represents a single species. The type specimen of *I. philippinensis* comes from Bataan, a peninsula in central Luzon, neither included in the area of the northern nor of the southern samples. Therefore, it is not yet clear whether one of these two lineages constitutes *I. philippinensis* in the sense of the protologue or whether the central Philippines might be home to a another, the typical, sublineage. More samples, including material from outside the Philippines presently included in *I. philippinensis,* need to be studied with combined morphological and molecular data to assess species boundaries within lineage IVa/B.

Lineage II/B3 comprises species centred in Mindanao and the Visayas, except for *I. salicifolia* which is widely distributed in the Philippines and also found in Borneo and Java. In this case, the present species concept may still apply, as the interspecies relationships in this subclade are unresolved. All members of lineage II/B3 apparently share a recent common geographic origin (note the length of the subtree roots in Fig. 1). Should further studies identify *I. salicifolia* in Malesia as an emigrant from the Philippines, it would underpin the key role of Mindanao as stepping stone for *Ixora* dispersal in the region [48]. Our sample identified as *I. longifolia* comes from Cebu and thus represents a typical member of the exclusively Philippine lineage II/B3. In the Philippines, this species is reported from southern Luzon, the Visayas, Mindanao and Palawan [35]. However, the type of *I. longifolia* was collected on the island of Honimoa (Moluccas, Indonesia) [49]. Further reports come from Borneo, Sumatra, and Amboina [35]. This may be an example for a species colonizing from the Philippines. However, because its area includes the Sunda Shelf (Borneo) and both western and eastern Wallacea (sensu [50]) and is thus extremely large for an *Ixora* species, detailed combined molecular and morphological analyses are necessary to establish whether the current concept of this species is valid.

### Biogeography

The signal from nuclear and plastid data is far too complex to allow for application of currently available methods of biogeographic inference. All current methods need a fully-, or at least well-resolved, ultrametric phylogenetic tree as input. Computation of such trees for the nuclear and plastid data, which would provide the necessary discriminating topology to obtain meaningful ancestral area reconstructions, is not feasible based on the available data. Signal strength is a limiting factor in the nuclear, and to aneven greater extent, the plastid, data. Deep incongruences prevent concatenating both data sets. In addition to primary nuclear-plastid incongruence, the genetic complexity in *Ixora* indicates phases of secondary reticulation (*I. brunonis,* possibly *I. acuticauda*) and incomplete lineage sorting (situation in lineages II+IV/B). These two evolutionary phenomena, not uncommon at the intra-generic level in plants, cannot be captured by a single phylogenetic tree. Thus, we used an alternative approach. The plastome is only maternally inherited and should be stronger geographically constrained than the nucleome, but less affected by early (during formation of species/lineages) or late (after formation of species/lineages) reticulation. Therefore, we assume that species with similar plastid signature come from the same area of origin. Similarity in the biparentally-inherited nucleome is taken as indication that two or more taxa are closely related in an evolutionary sense, and have not been isolated for a long time. It has been shown that in densely sampled, sympatric oak species speciation processes directly affect the nucleome, but not the plastome [51–55]. Two closely related species are more likely to hybridize and introgress, which will eventually lead to a homogenization of the nucleome but not necessarily of the plastome. Widespread species with species-diagnostic nuclear signatures can carry distinct plastid signatures (e.g. *Quercus coccifera* and *Q. ilex* [54, 56]). Thus, nuclear data will more likely reflect the (current) systematic affinity of an individual or species, whereas plastid data may reflect the provenance of the (mother) population (e.g. [55, 56] for oaks, and [57, 58] for Nothofagaceae).

The simplest explanation for the geographic distribution of nuclear and plastid lineages (Fig. 4) is that an originally South Asian-Indomalayan sister lineage (orange) of the exclusively Philippine lineage (red), migrated or expanded into the southern Pacific area, possibly in response to the expansion of earlier diverged other *Ixora* in that region (green and purple members of the African-Asian clade). Alternatively, assuming that the root in the plastid tree may be slightly misinformed (note the central placement of the purple, B-type, haplotypes in the MJ networks in Fig. 5), the divergence between the Pacific (orange) and African-Asian (blue, green) plastids effectively represents a geographic differentiation already between a (continental) South/Southeast Asian (green, purple) and Malesian-Australasian lineage (red and orange; Figs. 4 and 5). In this context, one should note the distinctness of the *trn*T-*trn*L spacers (Fig. 5), the most variable plastid marker included here with an overall divergence hindering alignment across all Ixoroideae. This is combined with lower divergence in the *rps*16 and *trn*L introns (and *trn*L-*trn*F spacer), plastid intron (and spacer) regions that can be straightforwardly aligned across all Ixoroideae (Additional file 1 and files provided in the electronic supplementary archive). Taken together, this could be indicative for a widespread common ancestor with a heterogenous plastome that was already starting to diversify due to genetic drift.

Under the primary assumptions (nuclear signal = systematic-phylogenetic affinity; plastid signal = geographic origin), the distinctness of both the nucleome and plastome of the exclusively Philippine lineage (red) suggests that this is the genuine (original) *Ixora* lineage of the Philippines, or at least a lineage originating and evolving in a different area to the rest of the genus. Thus, the modern mosaic of haplotypes found on the Philippines bears witness of several colonization waves by the African-Asian *Ixora* (lineage II+IV/B)*,* highlighted by the plastid signatures found in that lineage. Only the least derived haplotypes of this lineage (purple, light green in Fig. 5) – with respect to the *trnL* intron and downstream *trn*L-*trn*F spacer [trnLLF region] and *rps*16 intron and in comparison to the haplotypes of the Pacific-Philippine lineages – are found on Palawan. More derived types (dark green, violet; Fig. 5) are scattered across the archipelago. These haplotypes can also be found outside of the archipelago (circles in haplotypes networks in Fig. 5), indicating multiple colonizations.

Our study presents an opportunity to make several inferences about the biogeographical patterns and diversification of the Philippine *Ixora.* In the Philippines, four major colonization routes, or biogeographic umbilici [59] have been identified as entryways to parts of the archipelago that have never been connected to a mainland. One colonization route includes the eastern island arc involving Sulu Archipelago-Mindanao-Leyte-Samar-Luzon which is most likely the route followed by the species of lineage Ia/A, seeing as their distribution is recorded in these areas. Relatively long isolation from the rest of *Ixora* and its potential Asian-Pacific siblings, as well as small population size and areas, would explain its marked distinctness (strong genetic drift). Interestingly, lineage Ia/A (orange; Figs. 4 and 5) predominantly occurs in the northern Philippines and comprises species collected from the provinces of Aurora *(I. angustilimba*), Ilocos Norte (*I. bibracteata*), Zambales (*I. luzoniensis*), and Batanes island (*Ixora* sp. 3). This may reflect the fact that northern Luzon constitutes one of the geologically comparatively old parts of the Philippines that has undergone considerable northwestern movement during the Neogene [5]. Two species from eastern Visayas, *I. reynaldoi* and *I. silagoensis,* collected in Samar and Leyte, respectively, as well as the two samples of *I. bartlingii* are also nested within clade Ia/A. *Ixora bartlingii* is a widespread species found in most islands or provinces but never recorded from the island of Palawan.

The four clades containing Philippine species of lineage(s) II+IV/B are derived from a general Asian group. Thus, our data suggest (at least) four independent colonization events between Southeast Asia and the Philippines for *Ixora*. This reflects the dispersal mode of the genus, whose fleshy fruits are dispersed by understorey birds with usually limited ranges of action [60].

Palawan is playing a special role in improving our understanding of Southeast Asian biogeography. While the famous Wallace’s line [2] separates Wallacea from the Sunda-Region including the Philippines, Huxley’s line [61], separates the Philippines (except for Palawan) from the Sunda-Region, thus linking the island of Palawan biogeographically to Borneo. Recent analyses by Van Welzen et al. [50] revealed evidence for partitioning of Malesia into three instead of two regions: the western Sunda Shelf minus Java (Malay Peninsula, Sumatra, Borneo), central Wallacea (Philippines, Sulawesi, Lesser Sunda Islands, Moluccas, Java), and the eastern Sahul Shelf (New Guinea). However, Van Welzen et al. [50] treat Palawan as part of the Philippines, while its plate tectonic history identifies it as part of Sundaland [5]. In our study, we have included five of the eleven *Ixora* species occurring in Palawan. For lineage Ia/A, our results indeed support the separation of the island from the Philippines along the traditional Huxley’s line, because the only widespread species included in this clade, *I. bartlingii,* was never recorded from Palawan. In lineages II/B and IV/B, however, representatives from Palawan are involved in several radiations (Figs. 1 and 5). Nevertheless, the comparison of the corresponding subclades in the nuclear and plastid trees (Fig. 1), and the in-depth analysis of the Philippine plastid haplotypes (Fig. 5) converge to a rather simple hypothetical scenario. The lineage represented by nuclear clade II diversified in Southeast Asia, with a Malesian sublineage (*I. iteophylla*) reaching Palawan, and (re)colonizing from here the Philippines (subclade with B3 haplotypes; the widespread *I. salicifolia* is known from Palawan, but could not be included in our study), but also Indonesia (*I. otophora*). Bottleneck events while jumping into Palawan and the rest of the Philippines would explain the incomplete lineage sorting expressed in the plastid of lineage II/B, with one (non-Philippine) haplotype shared with members of the nuclear clade IV, while the other two are distinct, but of ambiguous phylogenetic affinity within clade B. One hypothesis could be that the founder populations were very small (a few seeds from the same parent population). Once established on Palawan they prevented further migration of their closest relatives. A similar situation may be that observed for *Hoya* on New Guinea*,* where the dominant Australasian lineage blocked the migration of two genetically more derived lineages except for a single sublineage each [62]. In contrast, in clade IVa, a clade with relatively underived B-type plastomes, the widespread species *I. philippinensis* and *I. macrophylla* are both reported from the island. The fact that the Palawan endemic *I. alejandroi* groups with the northern accessions of *I. philippinensis* (2, 5), and not with the *I. philippinensis* accession from Palawan in the southern group, indicates that, at least for this group of species, regular exchange between all Philippine islands, including Palawan, is still taking place. In its sister lineage IVb/B, the widespread species *I. cumingiana* is present on Palawan as well as on the other islands. Because our study does not include samples of these species from Palawan, their migration routes inside the Philippine archipelago remain to be investigated.

For the species of lineage(s) II+IV/B, our present results support the conclusion of Atkins et al. [11] in *Cyrtandra* (Gesneriaceae), who found that Palawan has both strong biogeographical ties with the other Philippine islands and, via Borneo, with the remainder of Sundaland. An increased sampling of the species recorded from Indonesia, particularly from Borneo, the most diverse area for the genus [24], will help to disentangle the supposedly multivalent role of Palawan for the biogeographic history of *Ixora*.

## Conclusions

For *Ixora*, the Philippines seem to constitute a crossroad where species from two major lineages in *Ixora*, the Pacific and the African-Asian one, have immigrated and subsequently radiated (Figs. 4 and 5). Our results further indicate that no secondary mixing has occurred between the two main lineages, as both nrDNA and cpDNA analyses suggest the same species groups. A more detailed study of lineage(s) II+IV/B, focusing on Asian material, is needed to understand the complex biogeographical patterns in *Ixora* inside the Malesian Region and adjacent continental Asia. Future studies should also include more populations, especially of species with wider distributions – be it on several islands or a presumed distribution on mainland Asia – to more finely resolve the phytogeography of the Philippine *Ixora* species.

## Methods

### Taxon sampling

Fieldwork was conducted in the Philippine islands from 2010 to 2013 in order to collect herbarium, alcohol and DNA material of Philippine *Ixora* species. We included 72 *Ixora* accessions (see Additional file 4), representing approximately 60 species of which 19 species are from other Asian countries and 24 species from the Philippines. Three Philippine specimens are potentially new species (pending formal description).

Following Mouly [31] and Alejandro et al. [17], three taxa from the tribes Aleisanthieae (*Greeniopsis multiflora* Merr. and *Aleisanthiopsis distantiflora* (Merr.) Tange) and Greeneeae (*Greenea corymbosa* K.Schum.) were chosen for the purpose of outgroup-rooting. Selection of suitable outgroup taxa was confirmed based on a full gene bank harvest (Additional file 1) for all four gene regions and guide trees optimized under ML (see Additional file 1). All other genera covered by data for all four gene regions are substantially more distant, in phylogenetic terms, from *Ixora* (see Figs. S1-1 to S1-5 in Additional file 1); and fairly difficult to align with *Ixora* (particularly in the case of the ITS region)*.*

### DNA extraction, amplification, sequencing, and alignment

Total DNA was extracted from dried material preserved in silica gel [63] following the protocols of DNeasy Plant Mini Kit (Qiagen, Germany). For the chloroplast regions, PCR mixes were made up to 25 μl each and contained 1.0 μl MgCl_2_, 2.5 μl 10 × PCR buffer, 2.0 μl dNTP, 1.0 μl of 10 μM forward primer, 1.0 μl of 10 μM reverse primer, 0.35 μl Taq DNA polymerase and 1 μl of total genomic DNA. Amplification of *rps*16 and trnT-F used the following PCR settings: initial melting phase of 2 min at 95 °C; followed by 35 cycles of 30 s at 95 °C, 1 min at 50–55 °C, and 2 min at 72 °C; and ended with a final extension phase of 7 min at 72 °C. The following sequence fragments were amplified: (1) the 3′ part of the 5′ external transcribed spacer of the nuclear-encoded 35S rDNA (ETS); (2) the complete ITS region comprising the internal transcribed spacers ITS1 and ITS2, and the 5.8S rRNA gene of the 35S rDNA; (3) the plastid *rps*16 intron; (4) the plastid *trn*T-*trn*L intergenic spacer (trnT-L); and (5) the adjacent trnLLF region comprising the *trn*L intron, the downstream (5′ or 1st) *trn*L exon, and *trn*L-*trn*F intergenic spacer. For primer pairs see Additional file 5.

PCR mixes for the nuclear regions were the same as for the chloroplast regions, except that 1 μl each of dimethylsulfoxide (DMSO) and bovine serum albumin (BSA) were added per 25 μl. The ETS amplification profile was: initial melting phase of 1 min at 97 °C; followed by 40 cycles of 10 s at 97 °C, 30 s at 55 °C, 30 s at 72 °C; and ended with a final extension phase of 7 min at 72 °C. The ITS amplification profile was: initial melting phase of 3 min at 94 °C; followed by 30 cycles of 1 min at 94 °C, 1 min at 52 °C, 1 min at 72 °C; and ended with a final extension of 7 min at 72 °C. Primers used for the amplification of nuclear [64–67] and chloroplast [68–71] DNA regions are listed in Additional file 4. PCR amplifications were run on a BIOMETRA thermocycler. All amplification products were cleaned using Qia-Quick PCR purification kit (Qiagen, Germany) and sent to LGC Genomics (Germany) for sequencing.

Forward and reverse sequences were edited and aligned using CodonCode Aligner version 4.0.4 (CodonCode Co., USA) and the consensus was exported in fasta format. The fasta files were aligned using SeaView version 4.0 [72] and the OPAL package [73] inside Mesquite [74] and the resulting alignment was corrected manually, following the recommendations of Kelchner [75]. All variable nucleotide positions were verified against the original electropherograms and final sequences uploaded to the European Nucleotide Archive (ENA).

### Phylogenetic analyses

Separate and combined analyses of the *rps*16, trnT-F (i.e. *trn*T-*trn*L spacer + trnLLF region), ITS and ETS matrices were performed using the Maximum Likelihood (ML) criterion as implemented in RAxML v. 8.1.20 [76] and Bayesian Markov Chain Monte Carlo (MCMC) inference [77] as implemented in the program MrBayes version 3.1 [78]. For ML, we run partitioned and unpartitioned analyses, treating each gene region as one partition. We used the standard command lines which allow for quick, simultaneous optimization of substitution model, topology and branch support via fast, non-parametric bootstrapping (options -f a; −x; −m GTRCAT). The number of necessary bootstrap (BS) replicates were determined by the extended majority rule criterion (−\# autoMRE [79]). MrBayes analyses used the same partitioning scheme as RAxML and two parallel runs with each one cold and three heated chains (standard set-up), 10^6^ generations, with every 1000^th^ topology sampled. Posterior probabilities (PP) are based on the final 5000 saved topologies from both runs. Investigation of differential support for alternative relationships can directly reveal significant incongruence between nuclear and plastid genealogies. Phylogenetic resolution at the intrageneric level is typically low, hence, the lack of (high) support should not be taken as indication for incongruence per se. Instead we regard only conflicting relationships as evidence for highly supported incongruence based on the nuclear vs. plastid data (BS_ML_ > 70, PP > 0.95; arbitrary thresholds following the common convention in plant phylogenetic literature). Bipartition networks [80], a special form of consensus networks [81] that use either the bootstrap replicate or Bayesian saved tree sample as input [82], visualized the differential support for alternative (competing) relationships (using SplitsTree v. 4.13 [83, 84]; option “count”). ‘Rogue’ taxa were further pinpointed using tanglegrams generated with Dendroscope 3 [85, 86]; the tanglegrams were also used to highlight congruence and incongruence of the nuclear and plastid topologies.

Local, in - detail differentiation patterns used median-joining (MJ) networks [87], computed with NETWORK v. 4.6 (Fluxus Technology Ltd) using default settings. MJ networks, a derivation of the more general median networks [88], were originally designed to study intraspecific (interpopulation) differentiation [87]. Here, they are used for their capacity to infer (parsimony-based) relationships based on low-divergent data subsets without forcing each sequence variant to the tip of a single tree (e.g. [89, 90]). The original matrices were first filtered for parsimony informative sites (to eliminate “satellite” sequence variants or singletons differing only by stochastic mutations from others) using PAUP* v. 4b10 [91]; occasional gaps (no prominent length polymorphism present) were treated as 5th base for the inference (gaps involving more than a single parsimony-informative site were treated as one mutational event for the final graphical representation). For MJ networks, the *trn*T-*trn*L and trnLLF region were treated separately to account for their different diversity.

## Additional files

Additional file 1: Details relating to the curation and analysis of the data harvested from gene banks to confirm selection of best-suited outgroups (PDF 1128 kb)

Additional file 2: Additional Figures **A**. Tanglegram showing maximum likelihood trees based on the nuclear (left) and the plastid (right) datasets including all sampled cultivated species (in red font). Branch numbers indicate bootstrap support values and posterior probabilities for selected branches. Clade designation as in main-text Fig. 1. **B**. Posterior probability (PP) networks based on the nuclear dataset with cultivars removed. Edge lengths are proportional to the PP of the corresponding taxon bipartition (branch in a tree). Clade designation and colouring as in main-text Fig. 2. **C**. Posterior probability (PP) networks based on the plastid dataset with cultivars removed. Edge lengths are proportional to the PP of the corresponding taxon bipartition (branch in a tree). Clade designation and colouring as in main-text Fig. 2 (PDF 688 kb)

Additional file 3: Overview and detailed list of ribo−/haplotypes resulting from the tree inferences and median-joining analyses (XLSX 103 kb)

Additional file 4: List of included specimens, with GenBank accession numbers (PDF 25 kb)

Additional file 5: List of used primers for amplification of nuclear and plastid target gene regions (PDF 15 kb)

## Abbreviations

BS: Bootstrap
BSA: Bovine Serum Albumin
cpDNA: chloroplast DNA
DAP: Distinct alignment pattern
DMSO: Dimethylsulfoxide
DNA: Deoxyribonucleic acid
ENA: European nucleotide archive
ETS: External transcribed spacer
ITS: Internal transcribed spacer
MCMC: Bayesian Markov Chain Monte Carlo
MJ: Median joining
ML: Maximum likelihood
nrDNA: Nuclear DNA
PAUP: Phylogenetic analysis using parsimony *and other methods
PCR: Polymerase chain reaction
PIC: Parsimony informative character
PP: Posterior probability
RAxML: Randomized axelerated maximum likelihood
*rps*16: Ribosomal protein S16
*trn*: tRNA gene
