# Supplementary material for: Ixora (Rubiaceae) on the Philippines - crossroad or cradle?
Source: BMC Evol Biol. 2017 Jun 7;17:131. doi: 10.1186/s12862-017-0974-3 (PMC5463362; doi:10.1186/s12862-017-0974-3)
Supplement: Supplementary file 2 — Additional Figures A. Tanglegram showing maximum likelihood trees based on the nuclear (left) and the plastid (right) datasets including all sampled cultivated species (in red font). Branch numbers indicate bootstrap support values and posterior probabilities for selected branches. Clade designation as in main-text Fig. 1. B. Posterior probability (PP) networks based on the nuclear dataset with cultivars removed. Edge lengths are proportional to the PP of the corresponding taxon bipartition (branch in a tree). Clade designation and colouring as in main-text Fig. 2. C. Posterior probability (PP) networks based on the plastid dataset with cultivars removed. Edge lengths are proportional to the PP of the corresponding taxon bipartition (branch in a tree). Clade designation and colouring as in main-text Fig. 2 (PDF 688 kb) [file 12862_2017_974_MOESM2_ESM.pdf]

Additional file 2: Additional figures. **A.** Reduced tanglegram showing maximum likelihood trees based on the nuclear (left) and the plastid (right) datasets including all sampled cultivated species (in red font). Branch numbers indicate bootstrap support values and posterior probabilities for selected branches. Clade designation as in main-text Fig. 1. **B.** Posterior probability (PP) networks based on the nuclear dataset with cultivars removed. Edge lengths are proportional to the PP of the corresponding taxon bipartition (branch in a tree). Clade designation and colouring as in main-text Fig. 2. **C.** Posterior probability (PP) networks based on the plastid dataset with cultivars removed. Edge lengths are proportional to the PP of the corresponding taxon bipartition (branch in a tree). Clade designation and colouring as in main-text Fig. 2.

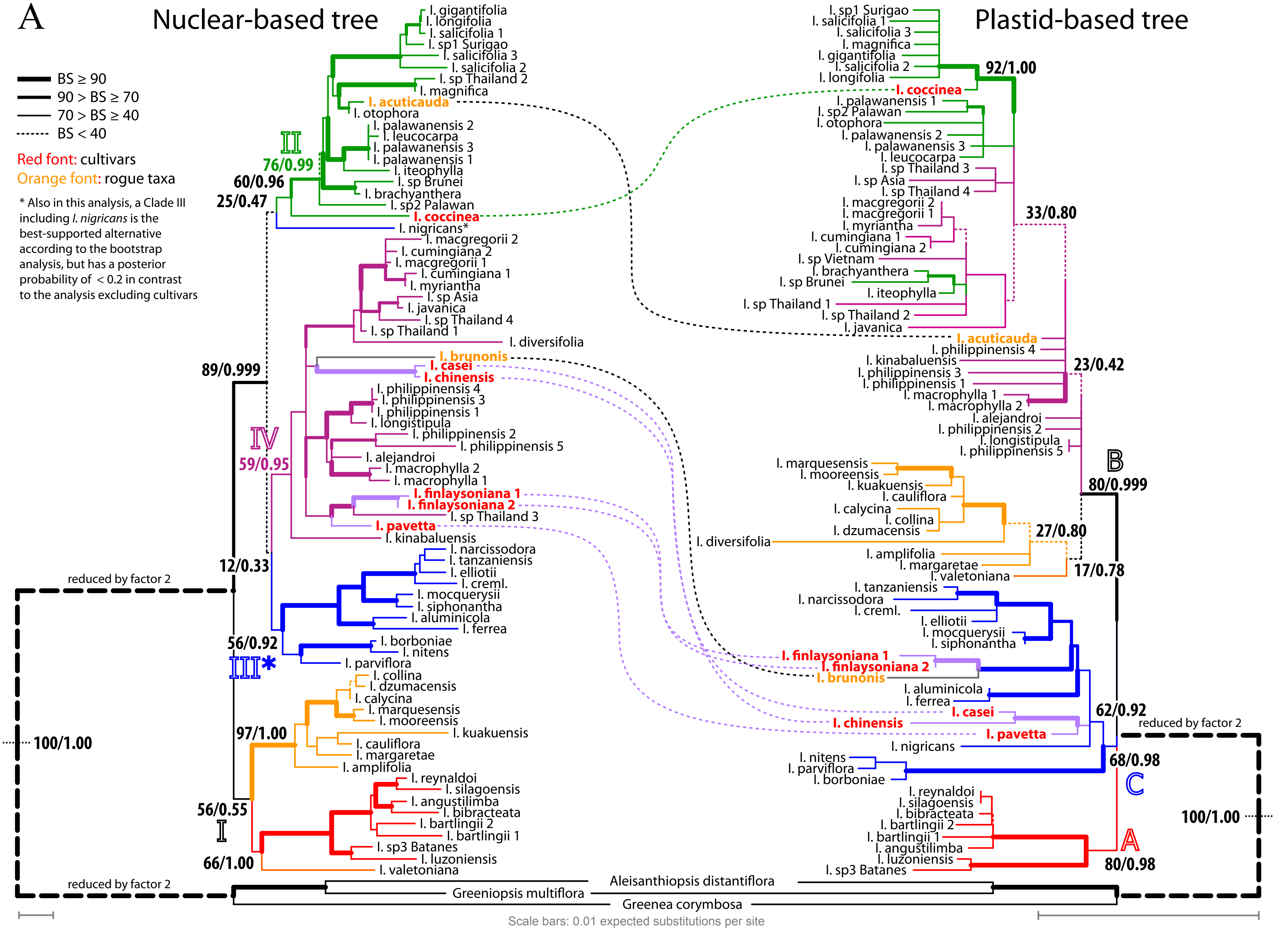

A
Nuclear-based tree
Plastid-based tree
BS ≥ 90
90 > BS ≥ 70
70 > BS ≥ 40
BS < 40
Red font: cultivars
Orange font: rogue taxa
* Also in this analysis, a Clade III including I. nigricans is the best-supported alternative according to the bootstrap analysis, but has a posterior probability of < 0.2 in contrast to the analysis excluding cultivars
I. gigantifolia
I. longifolia
I. salicifolia 1
I. sp1 Surigao
I. salicifolia 3
I. salicifolia 2
I. sp Thailand 2
I. magnifica
I. acuticauda
I. otophora
I. palawanensis 2
I. leucocarpa
I. palawanensis 3
I. palawanensis 1
I. iteophylla
I. sp Brunei
I. brachyanthera
I. sp2 Palawan
I. coccinea
I. nigricans*
II
76/0.99
60/0.96
25/0.47
I. macgregorii 2
I. cumingiana 2
I. macgregorii 1
I. cumingiana 1
I. myriantha
I. sp Asia
I. javanica
I. sp Thailand 4
I. sp Thailand 1
I. diversifolia
I. brunonis
I. casei
I. chinensis
I. philippinensis 4
I. philippinensis 3
I. philippinensis 1
I. longistipula
I. philippinensis 2
I. philippinensis 5
I. alejandroi
I. macrophylla 2
I. macrophylla 1
I. finlaysoniana 1
I. finlaysoniana 2
I. sp Thailand 3
I. pavetta
I. kinabaluensis
89/0.999
IV
59/0.95
12/0.33
I. narcissodora
I. tanzaniensis
I. elliotii
I. creml.
I. mocquerysii
I. siphonantha
I. aluminicola
I. ferrea
I. borboniae
I. nitens
I. parviflora
56/0.92
III*
reduced by factor 2
I. collina
I. dzumacensis
I. calycina
I. marquesensis
I. mooreensis
I. kuakuensis
I. cauliflora
I. margaretae
I. amplifolia
97/1.00
100/1.00
56/0.55
I. reynaldoi
I. silagoensis
I. angustilimba
I. bibracteata
I. bartlingii 2
I. bartlingii 1
I. sp3 Batanes
I. luzoniensis
I. valetoniana
I
66/1.00
reduced by factor 2
Aleisanthiopsis distantiflora
Greeniopsis multiflora
Greenea corymbosa
I. sp1 Surigao
I. salicifolia 1
I. salicifolia 3
I. magnifica
I. gigantifolia
I. salicifolia 2
I. longifolia
92/1.00
I. coccinea
I. palawanensis 1
I. sp2 Palawan
I. otophora
I. palawanensis 2
I. palawanensis 3
I. leucocarpa
I. sp Thailand 3
I. sp Asia
I. sp Thailand 4
I. macgregorii 2
I. macgregorii 1
I. myriantha
I. cumingiana 1
I. cumingiana 2
I. sp Vietnam
I. brachyanthera
I. sp Brunei
I. iteophylla
I. sp Thailand 1
I. sp Thailand 2
I. javanica
33/0.80
I. acuticauda
I. philippinensis 4
I. kinabaluensis
I. philippinensis 3
I. philippinensis 1
I. macrophylla 1
I. macrophylla 2
I. alejandroi
I. philippinensis 2
I. longistipula
I. philippinensis 5
23/0.42
B
80/0.999
I. marquesensis
I. mooreensis
I. kuakuensis
I. cauliflora
I. calycina
I. collina
I. dzumacensis
I. diversifolia
27/0.80
I. amplifolia
I. margaretae
I. valetoniana
17/0.78
I. tanzaniensis
I. narcissodora
I. creml.
I. elliotii
I. mocquerysii
I. siphonantha
I. finlaysoniana 1
I. finlaysoniana 2
I. brunonis
I. aluminicola
I. ferrea
I. casei
I. chinensis
I. pavetta
62/0.92
reduced by factor 2
I. nigricans
I. nitens
I. parviflora
I. borboniae
68/0.98
C
100/1.00
I. reynaldoi
I. silagoensis
I. bibracteata
I. bartlingii 2
I. bartlingii 1
I. angustilimba
I. luzoniensis
I. sp3 Batanes
A
80/0.98
Scale bars: 0.01 expected substitutions per site

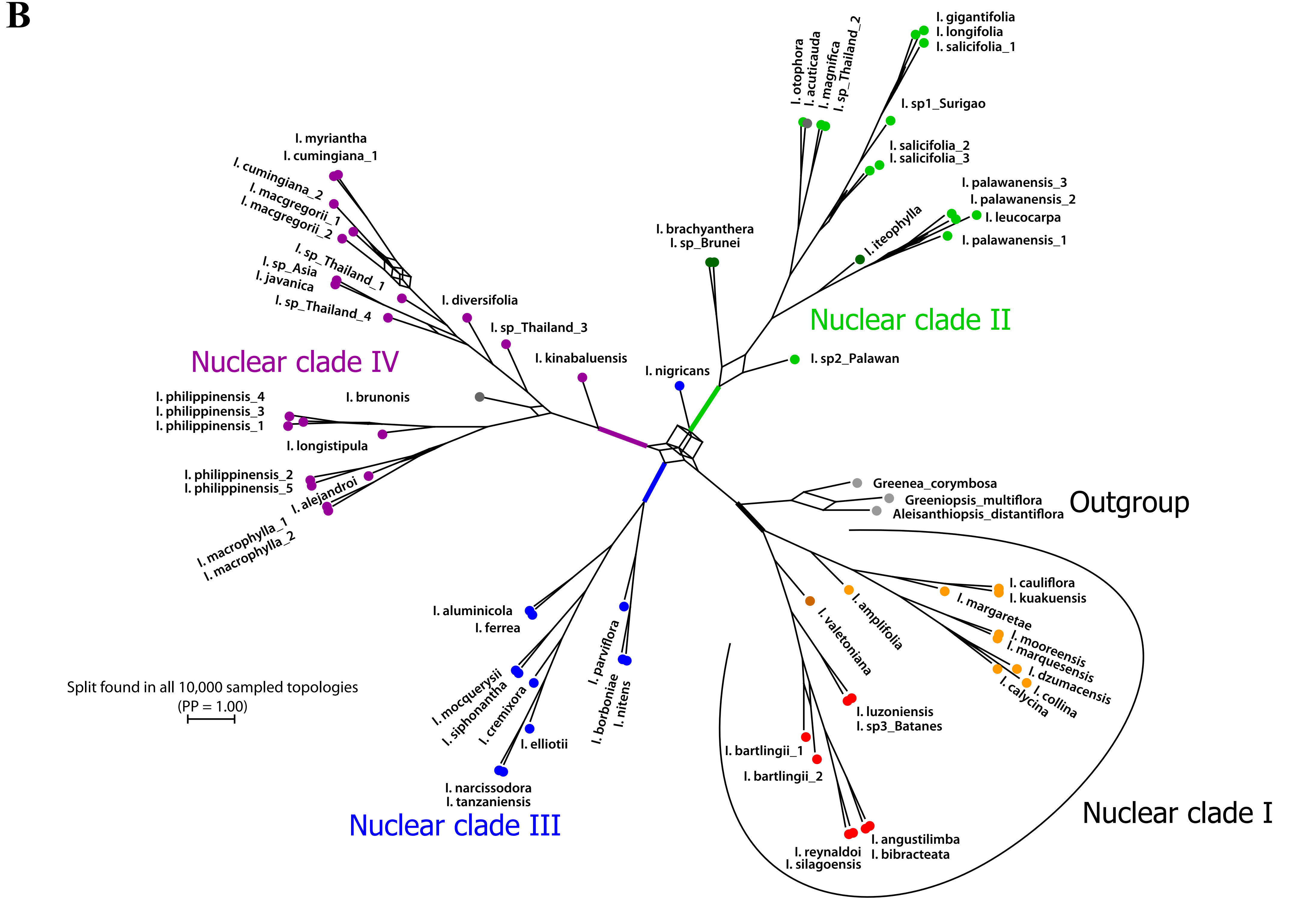
B
I. gigantifolia
I. longifolia
I. salicifolia_1
I. otophora
I. acuticauda
I. magnifica
I. sp_Thailand_2
I. sp1_Surigao
I. salicifolia_2
I. salicifolia_3
I. palawanensis_3
I. palawanensis_2
I. leucocarpa
I. iteophylla
I. palawanensis_1
I. brachyantheraI. sp_Brunei
Nuclear clade II
I. sp2_Palawan
I. nigricans
I. myriantha
I. cumingiana_1
I. cumingiana_2
I. macgregorii_1
I. macgregorii_2
I. sp_Thailand_1
I. sp_Asia
I. javanica
I. sp_Thailand_4
I. diversifolia
I. sp_Thailand_3
I. kinabaluensis
Nuclear clade IV
I. brunonis
I. philippinensis_4
I. philippinensis_3
I. philippinensis_1
I. longistipula
I. philippinensis_2
I. philippinensis_5
I. alejandroi
I. macrophylla_1
I. macrophylla_2
Greenea_corymbosa
Greeniopsis_multiflora
Aleisanthiopsis_distantiflora
Outgroup
I. cauliflora
I. kuakuensis
I. margaretae
I. amplifolia
I. valetoniana
I. mooreensis
I. marquesensis
I. dzumacensis
I. collina
I. calycina
I. luzoniensis
I. sp3_Batanes
I. bartlingii_1
I. bartlingii_2
I. angustilimba
I. bibracteata
I. reynaldoi
I. silagoensis
Nuclear clade I
I. aluminicola
I. ferrea
I. mocquerysii
I. siphonantha
I. cremixora
I. elliotii
I. parviflora
I. borboniae
I. nitens
I. narcissodora
I. tanzaniensis
Nuclear clade III
Split found in all 10,000 sampled topologies
(PP = 1.00)

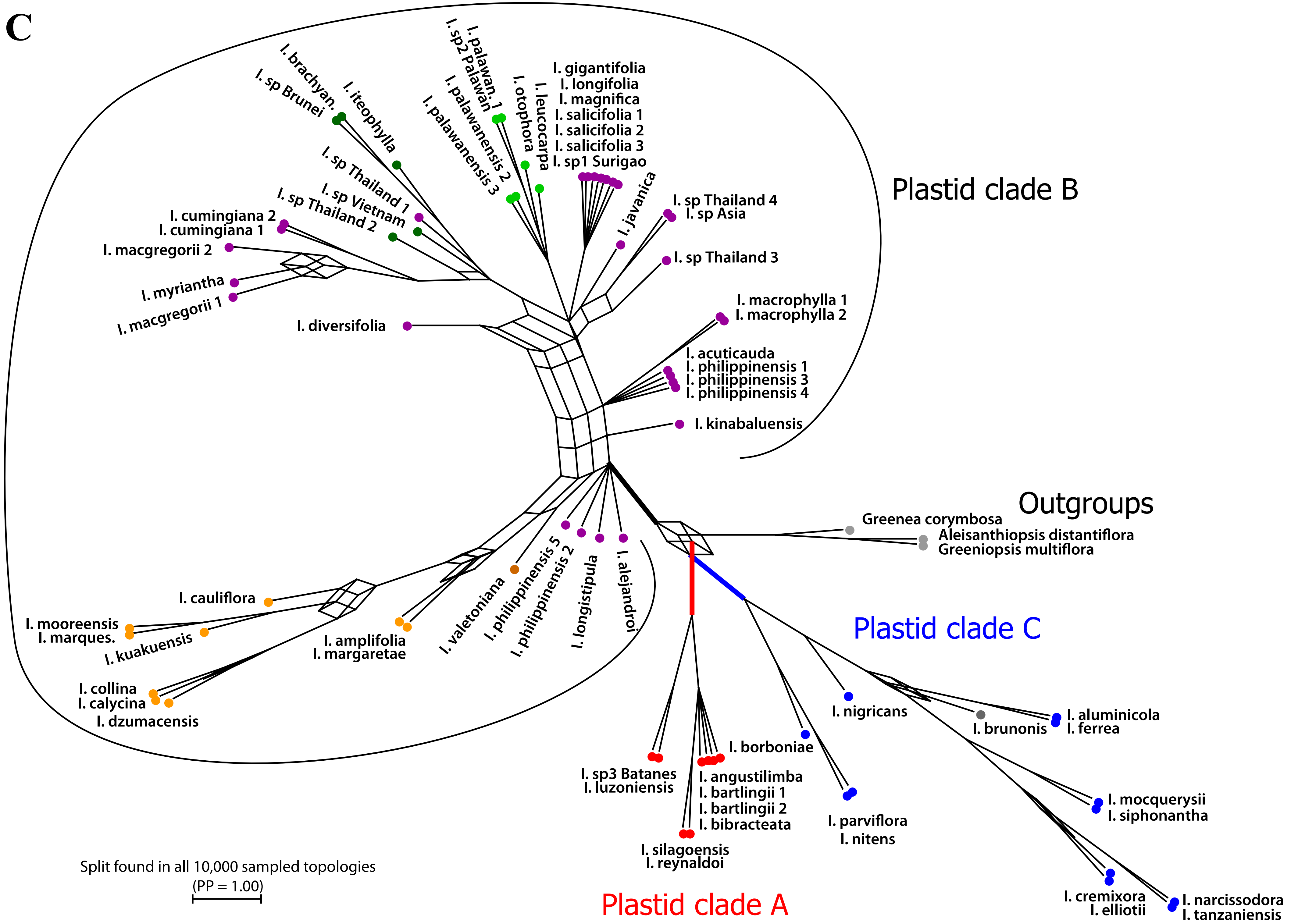
C
Plastid clade B
Outgroups
Plastid clade C
Plastid clade A
I. brachyan.
I. sp Brunei
I. iteophylla
I. palawan 1
I. sp2 Palawan
I. palawanensis 2
I. palawanensis 3
I. otophora
I. leucocarpa
I. gigantifolia
I. longifolia
I. magnifica
I. salicifolia 1
I. salicifolia 2
I. salicifolia 3
I. sp1 Surigao
I. javanica
I. sp Thailand 4
I. sp Asia
I. sp Thailand 3
I. sp Thailand 1
I. sp Vietnam
I. sp Thailand 2
I. cumingiana 2
I. cumingiana 1
I. macgregorii 2
I. myriantha
I. macgregorii 1
I. diversifolia
I. macrophylla 1
I. macrophylla 2
I. acuticauda
I. philippinensis 1
I. philippinensis 3
I. philippinensis 4
I. kinabaluensis
Greenea corymbosa
Aleisanthiopsis distantiflora
Greeniopsis multiflora
I. cauliflora
I. mooreensis
I. marques.
I. kuakuensis
I. collina
I. calycina
I. dzumacensis
I. amplifolia
I. margaretae
I. valetoniana
I. philippinensis 5
I. philippinensis 2
I. longistipula
I. alejandroi
I. nigricans
I. brunonis
I. aluminicola
I. ferrea
I. borboniae
I. sp3 Batanes
I. luzoniensis
I. angustilimba
I. bartlingii 1
I. bartlingii 2
I. bibracteata
I. silagoensis
I. reynaldoi
I. parviflora
I. nitens
I. mocquerysii
I. siphonantha
I. cremixora
I. elliotii
I. narcissodora
I. tanzaniensis
Split found in all 10,000 sampled topologies
(PP = 1.00)
