# Supplementary material for: Ixora (Rubiaceae) on the Philippines - crossroad or cradle?
Source: BMC Evol Biol. 2017 Jun 7;17:131. doi: 10.1186/s12862-017-0974-3 (PMC5463362; doi:10.1186/s12862-017-0974-3)
Supplement: Supplementary file 4 — List of included specimens, with GenBank accession numbers (PDF 25 kb) [file 12862_2017_974_MOESM4_ESM.pdf]

Additional File 4. Accessions sampled for the study.

Taxon; locality; voucher specimen (herbarium code); GenBank accession numbers (rps16 intron, trnT–F region, ITS, ETS; an n-dash denotes missing sequences; {} indicate separately sequenced trnT-L intergenic spacer and trnL-F region.

Aleisanthiopsis distantiflora (Merr.) Tange; Indonesia; *Kessler & al. 41* (P); EU817434, EU817453, JQ398731, FJ150425. ***Greenea corymbosa*** K.Schum.; Thailand; *Beusekom et al. 752* (P); AF242961, AF152657, – , FJ150435. ***Greeniopsis multiflora*** Merr.; Philippines; *Uy & Tandang 25007* (USTH); GQ981432, GQ981424, JQ398734, –. ***Ixora acuticauda*** Bremek.; Malaysia: Borneo; *Axelius 241* (S); –, {LT799443, LT799448}, LT799429, LT799434. ***Ixora alejandroi*** Banag; Philippines: Palawan; *Tandang 254886* (PNH); HG970792, HG970757, HG964391, HG970820. ***Ixora aluminicola*** Steyerm.; French Guiana; *Prévost 4160* (P); FJ150617, FJ150541, HG315441, FJ150436. ***Ixora amplifolia*** A. Gray; Samoa; *Takeuchi 7101* (L); LT799439, {LT799444, LT799449}, LT799430, LT799435. ***Ixora angustilimba*** Merr.; Philippines: Aurora; *Banag 11032* (USTH); HG323826, HG970758, HG964361, HG970821. ***Ixora bartlingii*** Elmer 1; Philippines: Samar; *Pinarok 121008* (USTH); HG970793, HG970760, HG964363, –. ***Ixora bartlingii*** Elmer 2; Philippines: Laguna; *Banag IM003* (USTH); HG323827, HG970759, HG964362, HG970822. ***Ixora bibracteata*** Elmer; Philippines: Ilocos Norte; *Arriola 13016* (USTH); HG970794, HG970761, HG964364, HG970823. ***Ixora borboniae*** Mouly & B.Bremer; Mascarene Is.; *Friedmann 3049* (P); FJ150685, FJ150609, HG315450, FJ150530. ***Ixora brachyanthera*** Bremek.; Brunei; *Ogata B 139* (L); LT799440, {LT799445, LT799450}, LT799431, LT799436. ***Ixora brunonis*** Wall. & G. Don; Thailand; *Larsen et al. 43463* (P); EU817446, EU817470, –, FJ150443. ***Ixora calycina*** Thwaites; Sri Lanka; *Tirvengadum et al. 18* (P); FJ150622, FJ150546, –, –. ***Ixora casei*** Hance; cultivated Taihiti, French Polynesia; *Mouly & Florence 348* (P); FJ150623, FJ150547, –, FJ150444. ***Ixora cauliflora*** Montrouz.; New Caledonia; *Mouly & Innocente 267* (P); FJ150624, FJ150548, HG315455, FJ150445. ***Ixora chinensis*** Lam.; cultivated Uppsala, Sweden; *no voucher*; FJ150625, FJ150549, –, FJ150446. ***Ixora coccinea*** L.; cultivated Uppsala, Sweden; *Bremer 2719* (UPS); EF205641, EU817464, AJ224826, FJ150448. ***Ixora collina*** Beauvis.; New Caledonia; *Mouly & Innocente 236* (P); FJ150626, FJ150550, HG315456, FJ150449. ***Ixora cremixora*** Drake; Madagascar; *Leeuwenberg 13879* (P); FJ150628, FJ150552, HG315458, FJ150451. ***Ixora cumingiana*** Vidal **1**; Philippines: Rizal; *Banag AN004* (USTH); HG970797, HG970764, HG964378, –. ***Ixora cumingiana*** Vidal **2**; Philippines: Pangasinan; *Banag BO001* (USTH); HG970796, HG970763, HG964366, HG970824. ***Ixora diversifolia*** R.Br.; Thailand; *Charoenphol et al. 3719* (P); FJ150629, FJ150629, –, –. ***Ixora dzumacensis*** Guillaumin; New Caledonia; *Mouly & al. 275* (P); FJ150630, FJ150554, –, FJ150453. ***Ixora elliotii*** Drake ex De Block, *sp. nov. ined.*; Madagascar; *Dumetz 1175* (P); FJ150632, FJ150556, HG315461, FJ150454. ***Ixora ferrea*** (Jacq.) Benth.; Puerto Rico; *Taylor 11693* (MO); EF205642, EU817465, HG315465, FJ150456. ***Ixora finlaysoniana*** Wall. & G.Don **1**; cultivated Tanzania; *Luke 9042* (S); EU821619, EU817466, –,

FJ150458. ***Ixora finlaysoniana*** Wall. & G.Don **2**; cultivated Philippines: Laguna; *Cua 1107c* (USTH); HG970798, HG970765, HG964367, HG970825. ***Ixora gigantifolia*** Elmer; Philippines: Surigao del Norte; *Banag SU012A* (USTH); HG970800, HG970767, HG964369, HG970826. ***Ixora iteophylla*** Bremek.; Malaysia; *Schaller & al. 3932* (P); FJ150640, FJ150563, –, FJ150469. ***Ixora javanica*** DC.; Laos; *Munzinger 119* (P); –, FJ150602, –, FJ150519. ***Ixora kinabaluensis*** Stapf; Malaysia: Sabah; *Beaman 10664* (L); LT799441, {LT799446, LT799451}, LT799432, LT799437. ***Ixora kuakuensis*** S.Moore; New Caledonia; *Munzinger 2180* (NOU); FJ150642, FJ150565, –, FJ150471. ***Ixora leucocarpa*** Elmer; Philippines: Palawan; *Banag I010* (USTH); HG970801, HG970768, HG964370, HG970827. ***Ixora longifolia*** Sm.; Philippines: Cebu; *Paraguison L010* (USTH); HG970815, HG970785, HG964388, –. ***Ixora longistipula*** Merr.; Philippines: Panay; *Chavez 12093* (USTH); HG970802, HG970769, HG964373, HG970828. ***Ixora luzoniensis*** Merr.; Philippines: Zambales; *Banag MA008* (USTH); HG970803, HG970770, HG964374, HG970829. ***Ixora macgregorii*** C.B. Rob. **1**; Philippines: Sorsogon; *Chavez 13315* (USTH); HG970818, HG970790, HG964394, HG970845. ***Ixora macgregorii*** C.B. Rob. **2**; Philippines: Sorsogon; *Chavez 13336* (USTH); HG970819, HG970791, HG964395, HG970846. ***Ixora macrophylla*** Bartl. ex DC. **1**; Philippines: Aurora; *Banag 11053* (USTH); HG323830, HG970771, HG964375, HG970830. ***Ixora macrophylla*** Bartl. ex DC. **2**; Philippines: Cebu; *Paraguison L009* (USTH); HG970804, HG970772, HG964376, –. ***Ixora magnifica*** Elmer; Philippines: Laguna; *Banag IM001* (USTH); HG970805, HG970773, HG964377, HG970831. ***Ixora margaretae*** (N. Halle) Mouly & B.Bremer; New Caledonia; *Mouly & Innocente 222* (P); EU817436, EU817456, –, FJ150426. ***Ixora marquesensis*** F.Br.; Marquesas Is.; *Mouly 504* (P); FJ150645, FJ150568, –, FJ150475. ***Ixora mocquerysii*** DC.; Madagascar; *Malcomber 2805* (MO); FJ150647, FJ150570, HG315487, FJ150477. ***Ixora moorensis*** (Nad.) Forberg; Society Is.; *Florence s.n.* (P); EU817441, EU817462, –, FJ150478. ***Ixora myriantha*** Merr.; Philippines: Davao del Sur; *Lemana BL005* (USTH); HG970806, HG970774, HG964379, HG970832. ***Ixora narcissodora*** K.Schum.; Kenya; *Luke 8324* (UPS); FJ150648, FJ150571, HG315488, FJ150479. ***Ixora nigricans*** R.Br.; Thailand; *Larsen et al. 43037* (P); FJ150650, FJ150573, –, FJ150482. ***Ixora nitens*** (Poir.) Mouly & B.Bremer; Mascarene Is.; *Friedman 2631* (P); FJ150684, FJ150608, HG315490, FJ150529. ***Ixora otophora*** Bremek; Malaysia: Sabah; *Beaman 10614* (L); LT799442, {LT799447, LT799452}, LT799433, LT799438. ***Ixora palawanensis*** Merr. **1**; Philippines: Palawan; *Banag LM002* (USTH); HG970808, HG970776, HG964380, HG970833. ***Ixora palawanensis*** Merr. **2**; Philippines: Palawan; *Medecillo MPM 471* (USTH); HG970807, HG970775, HG964381, –. ***Ixora palawanensis*** Merr. **3**; Philippines: Palawan; *Banag I008* (USTH); HG970799, HG970766, HG964368, –. ***Ixora parviflora*** Lam.; Mascarene Is.; *Lorence 1526* (P); EU817449, EU817473, AJ224840, FJ150533. ***Ixora pavetta*** Andrews; cultivated Uppsala, Sweden; *FTG 1738* (UPS); FJ150653, FJ150576, –, FJ150485. ***Ixora philippinensis*** Merr. **1**; Philippines: Davao del Sur; *Lemana BL006* (USTH); HG970809, HG970777, HG964385, –. ***Ixora philippinensis*** Merr. **2**; Philippines: Ilocos Norte; *Banag BU002* (USTH); HG970810, HG970778, HG964384, HG970834. ***Ixora philippinensis*** Merr. **3**; Philippines: Palawan;

Alejandro 12400 (USTH); HG970811, HG970779, HG964383, HG970835. ***Ixora philippinensis*** Merr. **4**; Philippines: Surigao del Norte; *Alejandro 11102* (USTH); HG970812, HG970780, HG964382, HG970836. ***Ixora philippinensis*** Merr. **5**; Philippines: Batanes; *Tandang DT548* (PNH); HG970813, HG970781, HG964386, HG970837. ***Ixora reynaldoi*** Banag; Philippines: Samar; *Banag SA004* (USTH); HG970814, HG970782, HG964390, HG970838. ***Ixora salicifolia*** (Blume) DC. **1**; Philippines: Leyte; *Banag 12026* (USTH); HG323828, HG970783, HG964371, HG970839. ***Ixora salicifolia*** (Blume) DC. **2;** Philippines: Leyte; *Banag SJ005* (USTH); HG323829, HG970784, HG964372, HG970840. ***Ixora salicifolia*** (Blume) DC. **3**; Philippines: Surigao del Sur; *Banag SU002* (USTH); HG323831, HG970786, HG964387, HG970841. ***Ixora silagoensis*** Manalastas, Banag & Alejandro; Philippines: Leyte; *Banag 12037* (USTH); HG323832, HG970787, HG964389, HG970842. ***Ixora siphonantha*** Oliv.; Madagascar; *Rabenantoandro et al. 944* (MO); FJ150661, FJ150584, HG315499, FJ150495. ***Ixora sp.*** **"Asia"**; Asia; *Martin 1314* (P); FJ150670, FJ150593, –, FJ150507. ***Ixora sp.*** **"Brunei"**; Brunei; *Malcomber et al. 2980* (MO); FJ150676, FJ150599, HG315500, FJ150516. ***Ixora sp.*** **"Thailand" 1**; Thailand; *Geesink 7226* (P); FJ150671, FJ150594, –, FJ150510. ***Ixora sp.*** **"Thailand" 2**; Thailand; *Larsen et al. 86KL14* (UPS); FJ150677, FJ150600, –, FJ150517. ***Ixora sp.*** **"Thailand" 3**; Thailand; *Vidal 5758B* (P); FJ150673, FJ150596, –, FJ150511. ***Ixora sp.*** **"Thailand" 4**; Thailand; *Vidal 5771* (P); FJ150672, FJ150595, –, –. ***Ixora sp.*** **"Vietnam"**; Vietnam; *Poilane 103* (P); FJ150675, FJ150598, –, –. ***Ixora tanzaniensis*** Bridson; Tanzania; *Luke 9304* (UPS); EU817447, EU817471, HG315502, FJ150520. ***Ixora valetoniana*** Mouly & B.Bremer; cultivated Bogor, Indonesia; *Ridsdale s.n.* (UPS); FJ150687, FJ150611, –, FJ150536. ***Ixora sp.*** **1 "Surigao"**; Philippines: Surigao del Norte; *Alejandro 11068* (USTH); HG970816, HG970788, HG964392, HG970843. ***Ixora sp. nov.*** **2 "Palawan"**; Philippines: Palawan; *Alejandro 12406* (USTH); HG970817, HG970789, HG964393, HG970844. ***Ixora sp.*** **3 "Batanes"**; Philippines: Batanes*; Tandang DT588* (PNH); HG970795, HG970762, HG964365, –.
