# Supplementary material for: Ixora (Rubiaceae) on the Philippines - crossroad or cradle?
Source: BMC Evol Biol. 2017 Jun 7;17:131. doi: 10.1186/s12862-017-0974-3 (PMC5463362; doi:10.1186/s12862-017-0974-3)
Supplement: Supplementary file 5 — List of used primers for amplification of nuclear and plastid target gene regions (PDF 15 kb) [file 12862_2017_974_MOESM5_ESM.pdf]

Additional File 5. Amplification primers for nuclear and chloroplast regions.

Region	Primer	Primer Sequence	Reference
ETS	18S-E	GCAGGATCAACCAGGTAGCA	[1,2]
	ETS-HL	GATCACAGCCTGAGCGGTG	
ITS	ITS1	GTCCACTGAACCTTATCATTTAG	[3,4]
	ITS4	TCCTCCGCTTATTGATATGC	
rps16	rpsF	GTGGTAGAAAGCAACGTGCGACTT	[5]
	rpsR2	TCGGGATCGAACATCAATTGCAAC	
trnT-F	A1	ACAAATGCGATGCTCTAACC	[6-8]
	I	CCAACTCCATTTGTTAGAAC	
	C	CGAAATCGGTAGACGCTACG	
	F	ATTTGAACTGGTGACACGAG	

1. Baldwin BG, Markos S: **Phylogenetic utility of the external transcribed spacer (ETS) of 18S-26S rDNA: congruence of ETS and ITS trees of *Calycadenia* (Compositae)**. *Mol Phylogenet Evol* 1998, **10**:449–463.
2. Razafimandimbison SG, Lantz H, Mouly A, Bremer B: **Evolutionary trends, major lineages, and new generic limits in the dioecious group of the tribe Vanguerieae (Rubiaceae): Insights into the evolution of functional dioecy**. *Ann Missouri Bot Gard* 2009, **96**:161–181.
3. White TJ, Bruns T, Lee S, Taylor J: **Amplification and direct sequencing of fungal ribosomal RNA genes for phylogenetics**. In: *PCR protocols: a guide to methods and applications.* Edited by Innis MA, Gelfand DH, Sninsky JJ, White TJ. San Diego: Academic Press; 1990: 315–322.
4. Urbatsch LE, Baldwin BG, Donoghue MJ: **Phylogeny of the coneflowers and relatives (Heliantheae: Asteraceae) based on nuclear rDNA internal transcribed spacer (ITS) sequences and chloroplast DNA restriction site data**. *Syst Bot* 2000, **25**:539–565.
5. Oxelman B, Liden M, Berglund D: **Chloroplast *rps16* intron phylogeny of the tribe Sileneae (Caryophyllaceae)**. *Plant Syst Evol* 1997, **206**:393–410.
6. Lantz H, Bremer B: **Phylogeny inferred from morphology and DNA data: characterizing well-supported groups in Vanguerieae (Rubiaceae)**. *Bot J Linn Soc* 2004, **146**:257–283.
7. Bremer B, Bremer K, Heidari N, Erixon P, Olmstead RG, Anderberg AA, Källersjö MK, Barkhordarian E: **Phylogenetics of asterids based on 3 coding and 3 non-coding chloroplast DNA markers and the utility of non-coding DNA at higher taxonomic levels**. *Mol Phylogenet Evol* 2002, **24**:274–301.
8. Taberlet PL, Gielly L, Patou G, Bouvet J: **Universal primers for amplification of three noncoding regions of chloroplast DNA.** *Plant Mol Biol* 1991, **17**:1105–1109.
