# Supplementary material for: Ixora (Rubiaceae) on the Philippines - crossroad or cradle?
Source: BMC Evol Biol. 2017 Jun 7;17:131. doi: 10.1186/s12862-017-0974-3 (PMC5463362; doi:10.1186/s12862-017-0974-3)

Gene bank harvest for outgroup data used in Banag et al.

Search strings

Rubiaceae: *txid24966[Organism] AND …*

Ixoroideae: *txid169618[Organism:exp] AND …*

Nuclear regions

ITS region[1]: *… "internal" [title]*

5'-ETS (2nd half): *… ("external" [title] OR "ETS" [title])*

Plastid regions

rps16 intron[2]: *… "rps16" [gene] NOT "rbcL" [gene]*

trnTLF region[3]: … ("trnT-trnL" [title] OR "trnL-trnT" [title] OR trnL [gene] OR "trnL-trnF" [title] OR "trnF-trnL" [title])

Harvest, prior-alignment, and curation

Using the programme GBK2FAS [1], we read out the meta-information associated with all Rubiaceae accessions stored in gene banks (per 28/02/2017; see File S1-1) to identify candidate genera for outgroups that cover all four gene regions included in our study. The substantially more variable 5'-ETS and ITS1 (and ITS2 to a lesser degree) are difficult to impossible to align above the subfamily level. Thus, we concentrated our search on the Ixoroideae. The NCBI GenBank flatfiles were transformed into FASTA-formatted sequence files using GBK2FAS, and aligned using mafft v.7.273 [2] using the --auto option, and --adjustdirection where needed. In case of the *trnTLF* region, an additional autoalignment was produced using the option --leavegappyregion to reduce the alignment length. MAFFT alignments were then visually expected using MESQUITE v.2.75 [3]; the ends were trimmed (removing primer sequences, blanking out potentially suboptimal data[4]) and obviously problematic data removed, and used to calculate quick guide trees to check for coherence of genera.

After the visual inspection and curation steps, the 64-bit executable of RAxML v. 8.0.20 [4] was used to infer maximum likelihood (ML) guide trees; branch support was established using the fast implementation of non-parametric bootstrapping with the number of necessary

---

[1] Search is non-comprehensive and will miss several sequences labelled e.g. "… ITS1, … ITS2, …"
[2] Gene banks include four accessions with the complete long single-copy unit (including the *rps16* gene), which were manually added to FASTA-files.
[3] Includes the *trnT-trnL* intergenic spacer, *trnL* gene (exons and intron), *trnL-trnF* intergenic spacer, and flanking parts of the *trnT* and *trnF* genes.
[4] A certain number of accessions in gene banks, particular those older than c. 10 years, show increased amount of mutations and single-nt gaps towards the ends of the sequences. These are likely editing artefacts, as becomes apparent by comparison with newer data on the same genus/species. 'Blanking out' means that potentially problematic data is replaced by the missing data symbol "?".

bootstrap (pseudo)replicates determined by the extended majority rule consensus bootstop criterion [5].

5'-ETS

Two sets of primers have been used to amplify the second half of the 5'-external transcribed spacer of the 35S rDNA. The *forward*-primers, ETS-Erit-F and ETS-HL are overlapping by apparently two nucleotides (nt), with ETS-HL located downstream of ETS-Erit-F. Both primers are not located in a generally conserved region of the 5'-ETS, as can be seen by comparison with the complete 35S rDNA of *Aslepias syriaca* JF312046 (same plant order, different family) that we used here as a template to define the ends of the rDNAs for both the 5'-ETS and ITS data sets. The initial portion of the accessions amplified using ETS-Erit-F can show up five or more point mutations when compared to the first 10 nt of primer ETS-HL. The *reverse*-primer 18S-E for both *forward*-primers is located in a highly conserved region including the last three nt of the 5'-ETS spacer and the start of the 18S rDNA (based on comparison with JF312046). For normalisation purposes, we capped the 5'-ETS data at both ends for the primer regions (ETS-Frit-F + ETS-HL; 18S-E).

General—The automatic alignment reveals three (four, if Ixoreae are counted) visibly distinct (5'-)ETS variants that differ mainly in GC/AT-contents and, partly, the general sequence structure. One (here addressed as 'ETS type C'; Additional file 1A) shows a completely different sequence that cannot be aligned with any of the other variants (neither in original orientation nor as reverse complement); the other two or three can be aligned with *Ixora* to some degree. 'ETS type C' represents a set of data generated for a study focussing on the Vanguerieae that used ETS and ITS data. The 5'-ETS regions of some of these genera have been sequenced for other studies (same primer set) and show the 'normal' ETS type (here addressed as 'ETS type A'; Additional file 1A). We performed MEGABLAST searches for an ETS type A and C accession of *Cantium*: the latter only finds hits with other ETS type C accessions of the same study (accession numbers EU….), whereas the ETS type A accession finds high identity with other genera of the tribe including accessions uploaded for different studies. Thus, we suspect that ETS type C is not at all representing the 5'-ETS of the nuclear-encoded 35S rDNA of Vanguerieae[5].

'ETS type B' collects accessions of *Mussaenda* (Mussaendeae) and *Emmenopterys* (classified as Ixorioideae incertae sedis), which are highly similar. Length-wise these sequences are comparable to those of *Ixora*; sequentially, they are very different and characterized by a high AT-content.

[5] MEGABLASTing of the *forward*-primer ETS-HL finds the following high-identity hits: (*i*) Rubiaceae (5'-ETS) accessions, where the used primers were not removed before uploading the sequences to gene banks; (*ii*) coding regions of various fungal genes; (*iii*) hits in the human genome. The complete 35S rDNA sequences available for Gentiales, the order including the Rubiaceae, are not among the hits. Also missing are accessions generated using the alternative 5'-ETS *forward* primer that supposedly is placed directly upstream (2-nt overlap) of ETS-HL.

Accessions with 'ETS type A' are more or less well alignable with *Ixora*. Most show, however, a higher GC-content and differ to various degrees from the Ixoreae 5'-ETS and a tendency for length-polymorphism (not seen in *Ixora* or the second sequenced genus of the Ixoreae, *Thouarsiora*). For instance, the Vanguerieae are characterized by a up to c. 40-nt long duplication/insertion pattern in the G-dominated region at pos. 320ff, not seen in members of any other tribe (consistently GTT in Mussaendeae + *Emmonopterys*, up to 10 nt-long motives in Gardenieae and tribes represented by a single accession, GTCGC in most Ixoreae)

Curation—Isolated, single-nt insertions in genera well covered by data were either blanked out (e.g. TCT in one accession when all others, of the same genus and other genera, show TT) or corrected for (e.g. TTT in one accession when all others, of the same genus and other genera, show TT) to facilitate the computation of representative consensus sequences. Corrections are indicated by lower-case letters in the alignments. Particularly in the case of multi-copy gene regions such as the 35S rDNA, isolated 1-nt length polymorphisms may be genuine or simply editing, amplification, or sequencing artefacts. Further curation included the adjustment of alignment of a single accession in parts with minor length-polymorphism to minimize number of gaps/characters; pos. 116–120 were left/right-aligned, pos. 147–150 were right-, pos. 154–156 left-aligned; pos. 264–268 (1-nt polymorphism in T-dominated motif in Vanguerieae) were left-aligned; (very) short sequence motives in all non-Vanguerieae were left-/right-aligned at pos. 320ff for consistency at tribal level; pos. 412–422 were essentially left-aligned to minimize gaps and inconsistent alignments at genus- and tribal level.

Problematic data—The 2nd half of the 5'-ETS seems to be relatively conserved at the genus level allowing straightforward identification of potentially mislabelled accessions.

Accession FN386412 (*Peponidium* sp. G. Gautier 4632) deviates visibly from other accession of this Vanguerieae genus, being sequentially more similar to accessions of *Fadogia* and *Multidenia.*

The accession representing *Rytigynia monantha* (FN386429) shows a conspicuous deletion in the 5' part of the sequenced strand not seen in any other species of the genus together with an increased amount of downstream point mutations and unique sequence motives. Lacking comparative data, it cannot be judged if this is a genuine signal or not.

ITS region

The accessions include either the complete strands of ITS1, 5.8S rDNA, and ITS2, or separate ITS1 or ITS2 sequences, with flanking rDNA gene regions (3' end of 18S, 5' end of 25S). One accession (*Coffea arabica,* EU650386) includes additional 607 nt from the 18S rDNA and 466 nt from the 25S rDNA. The 5.8S rDNA is highly conserved at the subfamily level as expected. The auto-alignment including all harvested data is gappy, which is partly due to included problematic data. Another issue maybe submission of raw sequences to gene bank, and issue with mixed signal from directly sequenced PCR products. Numerous single-nt indels relates to nt-repeats (e.g. AAA instead of AA in all or far the most other accessions) in

a single or very few sequences, which may be simple editing/sequencing artefacts. This is exemplarily true for older sequences of *Coffea* (e.g. AF542978ff). Overall, the auto-alignment worked well until the level of tribes, with exception of accessions of several Gardenieae including only the ITS1 (with no flanking rDNAs), where the end of the sequences was extended across the 5.8S for the last c. 20–30 nt. The ITS1 in this tribe is relatively length-polymorphic. Alignment between tribes can be ambiguous for parts the ITS1 due to strongly different sequence composition in generally length-polymorphic parts, mainly in the GC-dominated parts upstream and downstream of the putative ITS cleavage site (cf. [6]), an A-rich region found in all tribes.

Curation—The partly misaligned Gardenieae were re-aligned using MESQUITE's pairwise alignment tool. Individual ITS1 and ITS2 strands from the same voucher (given that information was provided in the gene bank accession) were then concatenated; and the alignment margins clipped for 18S and 25 rDNA portions covered by only a single to few sequences. As for the 5'-ETS, isolated single-nt insertions were blanked out or corrected for, and sequence ends trimmed. The artificial nature of such isolated, single-nt insertions is indicated by the fact that they do also occur in sequentially and structurally highly conserved parts (across angiosperms) of the rDNAs. According inconsistent alignments of single nt were adjusted. The ITS1 was quickly right-/left aligned along oligonucleotide motives found in all/most accessions to minimize the number of gaps. Regions of ambiguous alignment were excluded for the inference of the guide tree. In ITS2 the initial multiple-C-multiple-G-dominated motif, was right-/left-aligned and its shorter downstream counterpart left-/right-aligned (any alignment in this two LPR across tribes is to some degree ambiguous). Alignment also failed for the LPR at the end of the ITS2, the sequence region corresponding to the 3rd arm of the folded ITS2 [7], where substantial length-polymorphism (of up to c. 50 nt) and sequence divergence can be found between tribes. For instance, the Coffeeae are characterized by a completely different sequence than e.g. the Gardenieae or Pavetteae (placed in the same larger clade based on plastid data). Moreover, two different oligonucleotide motives within the same genus (*Coffea, Pavetta, Tarenna*) can be found.

Problematic data—Like the 5' ETS the ITS region appears to be generally conserved within accessions of the same Ixoroideae genus. Exceptions include *Coffea (arabica),* where a set of recently produced sequences (KP776610–KP776626) from areas cultivating coffee show a strongly deviating, AT-rich sequence. The 5.8S rDNA, a gene region sequentially and structurally conserved among all angiosperms, is AT-enriched and shows numerous deletions. These accessions are clearly not functional ITS copies.

A batch of old ITS2 sequences of *Coffea* (U6….) differ by numerous mutations from the general ITS2 type of the genus, including deviation patterns typical for gel-sequencer reads compared to later sequencing reads (cf. [8]). They may be due to suboptimal sequence reads and were removed from the alignment.

Accession DQ153639 (*Coffea heterocalyx*) is a chimera: the ITS1 shows the sequence characteristics of *Coffea* (*heterocalyx*), recombined with a *Tricalysia*-ITS2 (material sequenced by the same and other groups; both are genera of the same tribe).

Two of the three ITS accessions classified as Alberteae (KM592324, *Razafimandimbisonia*; KM592359, *Tennantia*) fall within the Pavetteae subtree. Vice versa, one sequence uploaded by the same authors representing the Pavetteae genus *Cladoceras* (KM592281) is placed as sister to the second of in total three Alberteae accessions (*Alberta*, AJ224842, uploaded by different authors) within a distinct subtree; base-per-base sequence comparisons confirm placements. The sequences appear to have been mixed up during uploading to the gene bank. Base-per-base comparison further shows that the sequence labelled as *Razafimandimbisonia* shows an ITS1 type exclusively shared by two genera of the Pavetteae (*Rutidea, Pavetta*); the accession uploaded as *Tennantia* shows a general type exhibited by several other genera of the Pavetteae, a association also supported by the available plastid data on the genus (placed always apart the Alberteae subtrees and included in the Pavetteae subtree).

The ITS1 and, in particular, the ITS2 sequence of *Alibertia bertierifolia* (AF183734f; Gardenieae) show numerous unique mutations and aberrant oligonucleotide motives in the LPRs. Best MEGABLAST-hits are with other accessions of the genus and *Borojoa*, although the score is comparatively low (~ 90% identity; other accessions of these genera have ≥ 95% identity. Lacking comparative data for the species, it cannot be judged if the sequence is genuine or aberrant.

The ITS2 sequence of *Borojoa* sp. Staahl 4410 (AF183781; Gardenieae) represents a degrading (pseudogenous) ITS2 copy (high AT-content); the ITS1 counterpart is inconspicuous.

One ITS2 sequence of *Duperrea pavettifolia* (KC533830; Gardenieae) deviates from the other six available for the genus and species, showing a highly unusual sequence. MEGABLAST gives a ≥ 98% identity with the Primulaceae *Ardisia*. The accession is mislabelled.

Three accession of *Gardenia* (KC533836, *G. jasminoides*, ITS2; JX856572, *G. resinifera*, ITS2; KP657895, *Gardenia* sp. GAR01, ITS1; Gardenieae) deviate from the general sequence pattern in the genus and tribe. KP657895 includes also a portion of the 5.8S not showing the consensus for the subfamily. Highest MEGABLAST-scores for KC533836 point towards an Araliaceae-ITS (*Panax* and relatives); whereas JX856572 is for the main part a Fabaceae-ITS (*Bauhinia*-group). Both accessions are mislabelled. The ITS1 part of KP657895 is relatively inconspicuous, and MEGABLAST finds other ITS accessions of the genus and tribe among the best hits; MEGABLASTing of the aberrant 5.8S part gives, however, no highly similar hits. The sequence was removed being of uncertain quality.

Accession KM592295 (*Gardenia rutenbergiana*; Gardenieae; de Block et al., unpubl.) shows a Pavetteae-ITS sequence and is placed in the according subtree (as sister of *Coptosperma graveolens*), apart from the subtree collecting all other accession of the genus. Notably, the

same authors uploaded a sequence as *Coptosperma borbonicum* (KM592283; Pavetteae), which is placed as sister to the *Gardenia*-clade. The sequences may have been mixed up during uploading to the gene bank (see above, accessions KM592359 and KM592281, uploaded by the same authors).

The only accession of *Hyptianthera* (KR532220, *H. stricta*; Gardenieae; ITS2 only) generated in course of the barcoding study of Huang et al. (2015, see above and footnote 5) shows an ITS2 variant found in the Vanguerieae *Canthium* and *Psydrax*. Plastid data on the genus by other authors confirms its association with the Gardenieae. The accession is clearly mislabelled.

Accession AJ224837 (*Oxyanthus*; Gardenieae) is placed within the Pavetteae subtree, but with a long-branch. Base-per-base sequence comparison shows that the sequence does not show an ITS1 variant characteristic for members of the Gardenieae vs. Pavetteae. MEGABLAST of the ITS1 region does not produce any clear hit, highest similarity scores (but < 90% identity) are found with members of the Coffeae as well as the Gardenieae, whereas the ITS2 gives best hits (~92%) exclusively with members of the Pavetteae. The scores are too low to unambiguously assign the sequence to a distinct genus or tribe. The plastid data available for the genus places it within the Gardenieae. More data are needed on the ITS region of the genus to rule out the possibility that the sequence is an artificial chimera or mislabelled.

Far the most *Randia* (Gardenieae) accessions are collected in the same subtree (which includes several other Gardenieae genera) except for AJ224831 (*R. moorei*) and AF183800 (*R. tessmannii*; ITS2 only). AJ224831 is placed within the *Aidia*-comprising subtree, AF183800 within the *Alibertia*+*Borojoa* subtree (all Gardenieae). Sequence comparison shows that AJ224831 is relatively similar to members of *Aidia* in the ITS1 and ITS2, but sequences are not identical. AF183800 differs by several unique point mutations from the general ITS2 variants found in *Alibertia-Borojoa*, but also shows clade-specific mutational patterns. There is no other data on these two species. Both, AJ224831 and AF183800, may be mislabelled or the species placed in the wrong genus. More data are needed.

The ITS1 accession representing *Stachyarrhena* (sp. indet., AF183803; Gardenieae) is misplaced in our guide tree simply due to the lack of data[6] (the position of its ITS2 counterpart, accession AF183804, sequenced from the same material, is the genuine one).

One of the two accessions representing *Pseudomussaenda flava* (KM592306; Mussaendeae) shows an ITS1 and ITS2 deviating from the general sequence found in other members of the tribe. High identity is found with sequences of another Ixoroideae tribe (Pavetteae), including sequences produced by the same authors. Vice versa, the sequence representing *Leptactina papalis* (KM592282; Pavetteae) is identified as a Mussaendeae by MEGABLAST search, with

[6] Owing to alignability, large parts of the ITS1 were not included when inferring the guide tree. Thus, any ITS1 variant distinct from the ITS1 portions of complete ITS sequences is likely to be misplaced in our guide tree

an ITS1 highly similar to the other accession representing *Pseudomussaenda*. Hence, we assume that the two sequences were mixed up during upload and relabelled them accordingly.

The last 500 nt of a batch of *Mussaenda pubescens* and *M. shikokiana* sequences (KC336498–KC339513; Mussaendeae) are off-alignment because they include non-ITS sequences at their 3' end, highly similar to identical to accessions uploaded to gene banks as "external transcribed spacer". The authors may accidently uploaded their full alignment accessions (the ITS part of the sequences is inconspicuous). The non-ITS data was blanked.

The two accessions (different authors) representing *Coptosperma graveolens* (KF805097 and KM592293) are placed apart from each other, and both outside the subtree collecting most accessions of this Pavetteae genus. With respect to the overall ITS diversity patterns, the accessions are not likely to come from the same species. With respect to their placement in the ITS guide tree (see also comment above on accession KM592283 and KM592295), their exact generic affinities remain unclear. Regarding the sequences of the ITS1 length-polymorphic motives not included for the inference of the ITS guide tree, KF805097 shows patterns shared by *Coptosperma* and other Pavetteae genera. On the other hand, the ITS1 of KM592293 shows length-polymorphic motives unique to one of the two ITS variants characteristic of the genus *Pavetta* according to the currently available data, indicative for mislabelling or placement of the species in the wrong genus. Another ITS accession labelled as *Coptosperma* sp. De Block al. 720 (KM592292) falls within a subtree comprising accessions of *Tarenna, Pavetta* (2nd ITS variant), and *Rutidea*. Also in this case, the length-polymorphic motives are indicative for a mislabelling or misassignment.

Accession AJ224825 representing *Leptactina platyphylla* (Pavetteae) shares the diagnostic features of one of the *Pavetta* ITS variants, and is accordingly resolved in the guide tree. The accession is mislabelled or the species requires taxonomic re-assessment.

Partly problematic accessions are AY555116 (*Sipanea biflora*) and KM048208 (*Platycarpum decipiens*): the first part of the ITS1 in both accessions is unlike any Ixoroideae, hence, poorly aligned. AY555116 also shows a distinct ITS2. No (highly) similar hits could be found using MEGABLAST; the sequence is likely an artificial chimera between an unknown source and a potentially genuine ITS. In case of AY555116, the remainder of the ITS1 finds best MEGABLAST-hits among accessions of *Randia* (different tribe; 90–95% identity), the ITS2 with *Acrosynanthus* (different subfamily) and *Pentagonia* (classified as Ixoroideae *incertae sedis*). The problematic part of KM048208 was blanked out. AY555116 was removed from the alignment.

Accession AF183801 (*Stenosepala hirsuta*, ITS1) shows many ambiguous base-calls indicating overlaying variants including length-polymorphism or is the product of a mediocre sequence run, which renders it unusable. Its counterpart shows a unique type, but most similar to the ITS2 of other genera of the Gardenieae according to MEGABLAST. Lacking comparative ITS data on the genus, the ITS2 accession was kept.

Accession KR532480 (Pavetteae: *Pavetta,* only ITS2) shows all sequence characteristics of the ITS2 of the Gardenieae *Aidia*. The accession, generated in course of a barcoding study (Huang et al., *PLoS ONE*, 2015), is obviously mislabelled.[7]

Two of the accessions of *Canthium horridum* (KP092781, KR531877; Vanguerieae) fall in the Gardenieae subtree comprising *Aidia* (among other genera). Both accessions are mislabelled or the original material was misidentified. MEGABLAST identifies KP092781 (complete ITS) as *Aidia*-ITS (97% identity to an *Aidia* sequence generated by the same authors) and the same applies to KR531877 (ITS2 only; ≥ 97% identity to KP092781 and *Aidia* accessions of the same and other authors). Most of the remaining ITS2 sequences of the species (KR531872–75) show a ITS2 sequence type with affinity to the Vanguerieae, however, a variant not seen in any other species of this genetically polymorphic and relatively well studied genus, but most similar to a possible mislabelled/misassigned ITS2 sequence (KP092782, labelled as *Catunaregam spinosa,* a Gardenieae; not further data on the genus available for comparison). The last accession labelled as *Canthium horridum* is KR531876 and shows a sequence type found in two more accessions generated in course of the same barcoding study and labelled as *Canthium simile* (KR531878/79) and with a *Psydrax*-type ITS2 (same tribe, genus genetically distinct from *Canthium* for all covered gene regions). All these problematic accessions have been generated for two barcoding studies dealing with tropical plants (Liu et al., *Divers. & Distr.*, 2015; Huang et al., *PLoS ONE,* 2015), which are prone to include erroneous (misidentified) data (see footnote 5). In the light of the amount of erroneously labelled accessions, we do not recommend to include any data produced by these two studies until the data have been verified using material that has been identified and verified by taxonomic experts on Rubiaceae.

The two accessions of *Pygmaeothamnus zeyheri* (Vanguerieae) are separated, one (JQ958001) placed within the *Cuviera* subtree (same tribe), the other (AJ315119) resolved as sister to the 3rd accession available for the genus (JQ958000; *P. chamaedendrum*). Base-per-base sequence comparison shows that JQ958001 differs from all *Cuviera* accessions by unique indel patterns in the ITS1 length-polymorphic regions, while sharing certain point mutations in contrast to the other two *Pygmaeothamnus* sequences. Further data are needed to test whether the two distinct sequence variants exhibited by JQ958001 and AJ315119 are indicative for misdetermination/-labelling or represent a genuine ITS polymorphism within the species/genus.

The accession representing *Rytigynia fuscosetulosa* (AJ315097) shows a *Vangueria*-type ITS. The same holds for the trnTLF sequence. Either the material has been misidentified, or the species should be included in *Vangueria.*

[7] Barcoding studies on tropical floras have become the main source of falsely labelled gene bank accessions. The failure to recognize a falsely labelled sequence in course of a barcoding study is particularly notable in this case as the same authors uploaded several more sequences to gene bank, which they assigned to the same species and that show inconspicuous ITS(2) sequences.

The two accessions of *Aleisanthiopsis* (*A. distantiflora*; JQ398731/32) differ from each other throughout the ITS1 and ITS2, with one being very similar to the ITS sequence of *Aleisanthus* (*A. rupestris*; sister genus). This is reflected in the position of both accessions in the guide tree. Further data on the two genera are needed to judge the representativeness of the two accessions.

The two accessions representing the genus *Henriquezia* (KM048206, KM048207) differ strongly in their ITS2 sequence, while showing the same unique ITS1 sequence. Both ITS2 variants appear to be unique within Ixoroideae; the motives are unusually long in the 5' ITS2 LPRs. MEGABLAST produces only four hits for the less aberrant sequence KM048207: with two of the four *Platycarpum* accessions in gene banks and the other *Henriquezia* accession, but the identity scores are far too low (~85%) to be decisive. Except for the other *Henriquezia*, no MEGABLAST hits are found for KM048206. Lacking comparative data, is cannot be judged whether these sequences (or one of them) are genuinely representing the genus (classified as Ixoroideae *incertae sedis*) or not, and both were tentatively included.

ITS2 accession KC533835 (*Wendlandia tinctorial*) is differs strongly from other accessions of the species (generated by the same or other authors). MEGABLAST identifies the sequences as *Euphorbia* (Malpighiales:Euphorbiaceae).

Table S1-1. Coherence of tribes represented by more than a single species in the ITS guide tree (meta-information as provided in gene bank flatfiles, following the current NCBI taxonomy tree; actual association may be different)

Tribe	Inclusive subtree	N(accessions[a])	N(genera)	Genera placed in according subtrees	Singletons	Separated by branches with BS ≥ 50
Alberteae	?[b]	3	3	0	3	0
Coffeeae	Yes	235	3	3	0	0
Condamineeae (incl. Hippotideae)	Yes[c]	44	15(+3)	6(+3)	9	0
Gardenieae	No[d]	187	26	9	12	1
Ixoreae	N/A[e]	8	1	1	0	0
Mussaendeae	Yes	152	3	3	0	0
Octotropideae	Yes	3	2	1	1	0
Pavetteae	Yes[f]	88	8	1	1	1
Posoquerieae	N/A[e]	13	1	1	0	0
Sipaneeea	No[f]	16	5	3	2	0
Vanguerieae	Yes	209	23	7	3	4

[a] Note that ITS1 and ITS2 accessions with the same voucher information were concatenated prior to analysis. Thus, the number of accessions is higher than the number of operational taxonomic units used during analysis.

[b] *Tennantia* placed within Pavetteae (as for *rps16* intron and trnTLF data). Accession labelled as *Cladoceras* may actually represent *Razafimandimbisonia,* and is placed as sister to the 3rd Alberteae accession within a *Bertiera* (Bertiereae)-Alberteae subtree.

[c] The subtree also includes the non-classified genus *Pentagonia.*

[d] The subtree including all Gardenieae also includes Alberteae, Bertiereae, Coffeeae, Octotropideae (only 3 accs representing three species of two genera), and Pavetteae

[e] Only species of one genus have been sequenced so far.

[f] One Sipaneae subclade (*Sipanea*) roots in this subtree, which is most likely a data artefact. The ITS regions (including the generally alignable portions but more so in the generally length-polymorphic regions) of the Sipaneae are most distinct from those in other Ixoroideae (and relatively distinct to each other) and the placement of the Sipaneae subclades (*Sipanea, Chalepophyllum+Dendrosipanea+Limnosipanea, Platycarpum*) to some degree random.

Chloroplast rps16 intron

The complete plastome of *Coffea arabica* (NC_008535) was used as template to define exon and intron regions. The *rps16* intron sequences in gene banks comprise nearly complete intron strands. The automatic alignment performed well, many original gaps are due to isolated 1-nt (pseudo)duplications, particularly at the start and end of accessions and their central part (zone of overlap of *uni* and *rev* reads; together with ambiguous base calls in some sequences in the same regions indicative for raw, unedited sequences uploaded to gene bank or mediocre sequence runs). Aside from a multiple T-motif in the central part of the intron, prominent length-polymorphism is restricted to intrageneric or -tribal indel variation (typically duplications). A complication for the alignment is that many accessions end in the part of the 3' region that includes some length-polymorphism at intra-tribal level.

Tree-wise, low taxonomic coherence (Table S1-1) despite relative high divergence can be found in the Vanguerieae.

Curation—The alignment was capped to include only the intron and truncated at the 3' end for sites defined for less than four accessions. The multi-A motif at pos. 83ff was right-aligned, the TA-motif at pos. 941 left-aligned, the multi-T and CT motives at pos. 261ff and 730ff right-/left-aligned. Few inconsistent alignment within tribes (single accessions) were corrected for, and, when possible, duplications (indels) moved to minimize gaps

Problematic or potentially problematic data—There are several accessions with sequences deviating from other sequences of their genus/tribe (e.g. AF242972, *Limnosipanea schomburgii,* is visibly strongly different from AF242973 of the same species, and later generated sequences of the same genus). The guide tree identified the following potentially mislabelled accessions.

AM117314 (*Heinsenia diervilleoides,* Gardenieae) differs by a number of point mutations in all spacer regions from the other two accessions of the species. The guide tree resolves it as closely related to members of *Coffea* (Coffeae)

Accessions representing the two species of *Aoranthe* are separated across the tree. The downstream 3' half-sequence AF200977 of *A. castaneofulva* is placed within the *Genipa* subtree, a short 5' fragment (AF200978, only 153 nt, same voucher) unresolved (placed in the

Oxyanthus subtree, critical branches without support); whereas the two accessions of the second species, *A.* (complete intron) are identical (they are also from the same voucher, and probably duplicates of the same sequence) and resolved as sister of *Massularia.*

Accessions representing the genus *Kutchubaea* are sequentially different, accessions of *K. semisericea* (identical, same voucher, probably duplicates of the same sequence) are resolved as sister to *Ibetralia*, whereas that of *K.* sp. Rodriguez 828 is genetically distinct from all other members of its subclade. The latter sequence shows an increased amount of unique mutational patterns, including single-nt duplicates. More data is needed to decide whether these are genuine patterns and not just (lack-of) editing artefacts.

The two accessions representing the genus *Sherbournia* (AF201017, *S.* sp. Cable 1046; KF964993, *S. calycina*) do not match up, and are accordingly placed in (phylogenetically) distant parts of the Gardenieae subtree (*S.* sp. is most similar to accessions of *Randia p.p., Kuchumennia,* and *Rothmannia*; *S. calycina* to accessions of *Oxyanthus*). Critical branches of the Gardenieae backbone have however little support and further data on the genus is needed.

Two *Randia* accessions (AJ320087, *R. schumannia*; KT218875, *R. maculata*) are placed apart from the subtree collecting all other accession of the genus. Sequentially they are inconspicuous. Failure to resolve their position may simply be due to limited signal supporting the genus (*Randia* sequences differ only by very few, not conserved at the genus-level, mutations from the consensus of most Gardenieae).

Despite from the same voucher, accessions of *Stenosepala (S. hirsuta*; AF201018+AF201019, KF964996) differ in their length-polymorphic motives in the 5'-part of the spacer (otherwise they show similar mutational patterns). The sequence of the newer accession in this part fits overall better to the general pattern observed for the subclade including *Stenosepala.*

Two of the *Schizenterospermum* (Pavetteae) accessions (KM592250, KM592251) group with *Coptosperma*, the third (KF964989) with *Paracephaelis.* Sequence-comparison reveals that the sequences of all three genera are very similar (including motives in length-polymorphic regions, LPR) and not diagnostic at the genus-level.

The two accession representing *Polysphaeria* (classified as Octotropideae) are visibly distinct. The newer one groups with other accessions of the same tribe (KF964975; *P. lanceolata*), whereas the older (AF201011; *P.* sp. Groves 529-129) is resolved as sister to *Afrocanthium* in the Vanguerieae subtree, indicating a misdetermination/contamination.

One accession labelled as *Limnosipanea schomburgkii* (AF242972) shows the rps16 intron sequence of the type genus of the Sipaneeae (likely an upload error; the second sequence of the species has been uploaded as AF242973 by the same author)

Table S1-2. Coherence of tribes represented by more than a single species in the rps16 intron guide tree (meta-information as provided in gene bank flatfiles, following the current NCBI taxonomy tree; actual association may be different)

Tribe	Inclusive subtree	N(accessions)	N(genera)	Genera placed in according subtrees	Singletons	Separated by branches with BS ≥ 50
Alberteae	No[a]	8	5	3	2	0
Coffeeae	Yes[b]	26	6	3	2	0
Condamineeae	Yes[c]	44	15(+4)	12(+3)	2(+1)	0
Gardenieae	No[d]	205	75(−2)	33	20(−2)	5
Ixoreae	No		2	2	0	0
Mussaendeae	Yes	13	6	1	6	0
Octotropideae	No[d]	22	10	5	4	0
Posoquerieae	Yes[e]	13(+7)	2(+2)	1(+2)	1	0
Pavetteae	Yes[d,f]	121(+2)	10(+2)	4	1(+2)	1
Sabiceeae	Yes	74	4	0[g]	1	0
Sipaneeea	Yes	19	5	2	3	0
Vanguerieae	Yes	29	12	4	3	4

[a] *Airosperma* (*trichotomum*; position needs further study) and *Tennantia* (*sennii*; 2 accs; included in Pavetteae) placed far apart.

[b] The subtree includes also *Bertiera* (only representative of Bertiereae), but not *Petitocodon*

[c] The subtree includes a clade collecting genera classified as Hippotideae and two *incertae sedis* genera (*Pentagonia, Tammsia*)

[d] Includes *Aulacocalyx* (only representative of Aulacocalyceae) as part of a Gardenieae subtree; Coffeeae and Pavetteae are nested insided the subtree collecting all Gardenieae. *Cremaspora* and genera classified as Octotropideae are collected in the same subtree (unsupported), including Gardenieae singletons.

[e] The two Posoquerieae are placed in a subtree with *Henriquezia* (*incertae sedis*) and *Platycarpum* (classified as Sipaneeae)

[f] The subtree includes *Canthiopsis (odorata)* and *Pelagodendron (vitiense)*, classified as Gardenieae.

[g] The *rps16* intron is not diagnostic for three of the four sequenced genera. Sequences of *Sabicea, Stipularia* and *Pseudosabicea* are very similar to identical.

Chloroplast trnTLF region

This region includes the *trnT*-*trnL* and *trnL*-*trnF* intergenic spacers, with the *trnL* gene (5' and 3' exon and intron) in between. The complete plastome sequences were used as template to define the intergenic spacer, intron and exon regions. Some accessions cover nearly the entire range (four accessions include also the downstream *trnF*-*ndhJ* intergenic spacer), others only the *trnT*-*trnF* intergenic spacer, or the *trnL* intron and downstream (5') exon plus *trnL*-*trnF* spacer. The automatic alignment using MAFFT's default settings appears to be consistent for the most part; they high proportion of gaps (total original alignment length: 4596 characters) relates to single or few sequences showing single-nt or oligonucleotide duplications (Fig. S1-1). Only few indel patterns are shared by accessions representing more than a single genus.

Parts of the *trnT*-*trnL* spacer are difficult to align across all tribes (sometimes within a tribe) due to prominent length-polymorphism (>50 nt difference in length). Within the *trnL* intron,

length-polymorphism is restricted to multiple-A(-T) motives and mostly isolated duplication events. Length-polymorphism is more prominently developed in the *trnL-trnF* spacer, but does not reach the level exhibited by the *trnT-trnL* intergenic spacer. The most prominent length-polymorphic feature is a 133-nt long duplication found only in last third of the *trnL-trnF* spacer of a single accession (AF201046; representing the genus *Agouticarpa*), replacing the usual sequence (up to xx nt-long) seen in all other taxa (to reduce alignment length, the duplication has been removed, its position indicated by missing data strand).

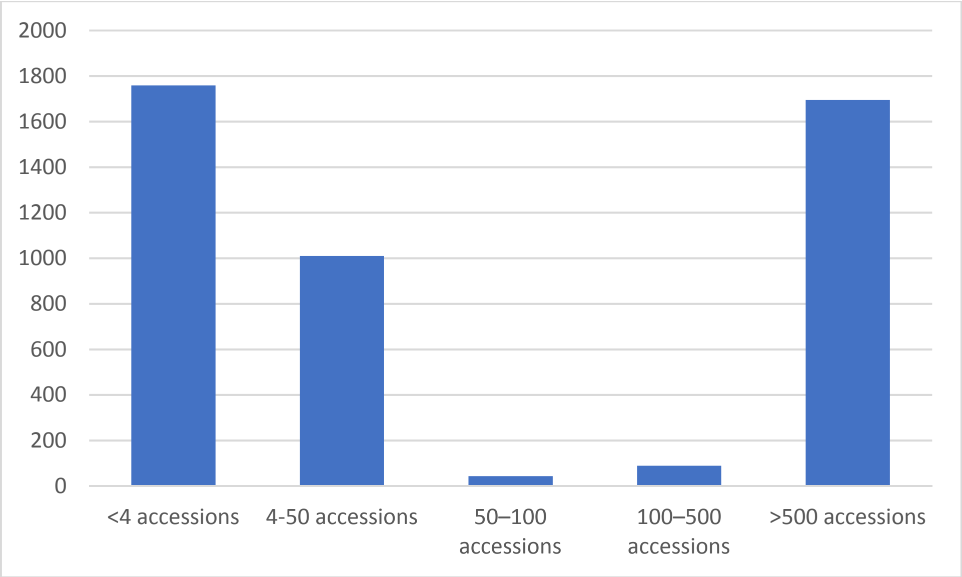

Fig. S1-1. Number of sites defined for a given number of accessions; values based on the unmodified automated alignment. Note the high number of sites defined for less than four accessions: many indels are restricted to a single or few accessions (total number of accessions: 1420; total number of characters: 4596)

Curation— The alignment was clipped to only include the intergenic spacers and the *trnL* gene region (exons and intron). Two *trnL* accessions of *Coffea* were out of alignment and re-aligned using MESQUITE's pairwise alignment tool. Occassional inconsistent alignments (within genera/tribes) was corrected for, and positions of duplications/insertions were adjusted in some cases. Pos. 431–477, … were left-/right-aligned, and the TA-dominated central part of the *trnT-trnL* spacer partly realigned using the longest motive(s) as template(s). Within the *trnL*, the multiple-A or multiple-T motives (pos. 1851ff, pos. 1929ff, pos. 2196ff, pos. 2216ff) were left- or right-aligned, respectively. In the *trnL-trnF* spacer the C/T-dominated motives at pos. 2512–2523, 2974–2987, and 3025ff were right- and left-aligned, respectively, and inconsistent alignment corrected for in the C/T-motif at pos. 2544–2560. Alignment of the generally length-polymorphic central part (pos. 2700–2803; some sequence patterns difficult to align across tribes) was adapted to minimize gaps.

Problematic or potentially problematic data identified using the guide tree—There are no accessions deviating visibly too strongly from accessions of their congeners. The guide tree includes a number of relatively prominent mid-level branches without BS support, which is an indication for internal signal conflict and may be partly due to the gappyness of the analysed matrix and non-alignability of length-polymorphic regions in the *trnT-trnL* spacer across all Ixoroideae. As for the *rps16* intron, the tribes as provided in the accessions' meta-information, can be coherent or not. At the genus level, there are more genera not resolved as exclusive clades than in the case of the *rps16* intron. Nonetheless these limitations, some accessions can be considered to be placed outside or far apart their expected clades and are listed in the following.

Accession KT218890 (*Alberta* sp. Tosh et al. 263; Alberteae) is virtually identical to accessions labelled as *Razafimandimbisonia*, a genus more recently erected within the same tribe and characterized by highly diagnostic accessions (long, well-supported root branch) and needs to be relabelled in analogy to the second accession from the same voucher (KM592145, *Razafimandimbisonia humblotii*).

Accession AM999485 (*Empogona coriacea,* Zambia, Coffeeae) is virtually identical to the main group of *Tricalysia* accessions from Madagascar and (continental) eastern Africa. In both genera, other haplotypes are present, which are not collected in the according subtree but scattered across the subtree that includes all Coffeeae genera except for *Coffea* and *Peticocodon*.

One *trnL* intron accession of *Tricalysia* (Coffeeae; KC479305, spec. indet., no voucher information) is placed within the *Ixora* (Ixoreae) subtree. It is clearly mislabelled and shows all characteristics of a *trnL* intron sequence the latter genus.

The accession representing *Xantonnea,* a genus classified as Octotropideae in the NCBI taxonomy tree, is deeply nested within the Coffeeae subtree. The genus may be wrongly classified (the genus has not been sequenced for any of the other here included gene regions).

One accession representing *Cremaspora (triflora;* KT218906; *trnL-trnF* spacer only) is placed outside the well-supported (high BS support, long root branch) *Cremaspora* clade and nested within the sister clade collecting most Octotropideae. Investigation of the alignment reveals that both tribes share the same basic *trnL-trnF* sequence with only random (stochastic) point mutations differentiating single accessions from all others in this region, and that the placement of KT218906 is simply a missing signal artefact.

One of the three accessions representing *Aidia* (Gardenieae; AF201028, *A. micrantha, trnL-trnF* only) is placed apart from its congeners. The situation here is similar to the one before: In contrast to the other two accessions, which show a variation at two sites from the tribal consensus, AF201028 simply shows the basic *trnL-trnF* sequence found in many other accession of the same tribe.

Another data coverage artefact is the disparate placement of accessions of *Atractocarpus* (Gardenieae). The accession with fewer sites covered form not part of the *Artractocarpus* clade. The long terminal branch of *Atractocarpus merikin* (KC428450) is due to an increased amount of unique point mutations, not found in any other member of the tribe, and usually linked to same nucleotide duplications (e.g. AGG or TGG instead of AAG or TTG in all other accessions). The latter could be indicative for a mediocre sequence run.

Accessions of the Gardenieae *Atractogyne (gabonii)* are also placed in different clades (all deeper branches with low or no BS support). Also here the *trnL-trnF* accession (355 nt-long) is placed apart the longer accessions (884 and 1704 nt-long). The same can be observed for *Catunaregam spinosa* (AF201038), *Porterandia* (AF201059), and possibly accounts for the scattering of accessions of *Rothmannia* (AF201062, AY555094, M117384; accessions differ also by isolated duplication patterns at pos. 2587ff, 2675ff, and 2992ff). The latter also applies to the two accessions (one complete strand, one *trnL-trnF* spacer) assigned to *Sherbournia* (KF965262, AF201064).

In case of *Morelia senegalensis* (Gardenieae; AF201055) the misplacement in the *Mitriostigma* clade (same tribe) is not only due to the covered segment (*trnL-trnF*) but also deviations in the sequence such as unique point mutations and mutational patterns seen in *Mitriostigma*. The same material (voucher Thomas 1915 [S]) has been re-sequenced for the entire strand, which provided a sequence nearly identical to that of its congener. The older data from the voucher is apparently not representative (possibly mixed/contaminated), and should be discarded.

One *trnL-trnF* accession of *Oxyanthus* (*O. racemosa*, AF201057) is placed outside the genus' clade. This is not a missing data artefact but a signal resolution issue. The *trnL-trnF* of *Oxyanthus* is very similar to that of *Mitriostigma*. Both genera show a few mutational patterns that separate them from all other Gardenieae, and differ by two point mutations (pos. 2572, 2809) and indel (pos. 2781–2785) in the *trnL-trnF*. With respect to those mutationals pattern, the *O. racemosa* accession is a mix sharing the single point mutations with its congeners, but showing the indel motive also found in *Mitriostigma,* resulting in its placement proximal to the root of the clade including both genera.

The accession representing *Randia maculata* (KT218940; *trnL-trnF* only) deviates from the genus consensus and is therefore placed in a different part of the Gardenieae (+ other tribes) subtree (resolved as sister to a *trnL-trnF* accession of *Rothmannia*). The genus was also not resolved with other gene regions, hence, it cannot be judged if the data are genuine or not. The same hold for the second deviating accession (KC428594, *R. tuberculosa*; *trnL* intron + *trnL-trnF*).

One *Ixora finlaysoniana* accession (complete strand; Ixoreae) is placed outside the *Ixora* clade. The reason for this is that this accession lacks some of the point mutations that characterize the genus and separate it from its putative sister genera such as *Greenea*. In

addition, it shows point mutations and duplication motives that are found in the other two accessions of the species. The accordingly conflicting signal is represented by its relatively long terminal branch and its position inbetween *Greenea* and *Ixora*. It cannot be judged if the signal is genuine or not.

Accession KT218938 (*Pseudomussaenda* sp. Dessein 1422; Mussaendeae) is virtually indistinct from sequences of *Mussaenda*, which are consistently placed in the sister clade of *Mussaenda* (both clades highly supported, BS_{ML} = 93/100). A misdetermination of the sequenced material cannot be ruled out.

One accession of *Schizenterospermum* (KF965257; *Sch.* sp. Davis 2580; Pavetteae) groups with *Paracephaelis* (moderately supported, BS_{ML} = 57, subclade within the Pavetteae subtree), whereas the other two accession of the genus fall within one of the *Coptosperma* subclades. Sequence comparison shows that KF965257 differs from the other two sequences at positions where *Paracephaelis* differs from the Pavetteae consensus. Also here, a misdetermination of the sequenced material cannot be ruled out. The same applies to one accession labelled as *Rutidea* (DQ180590, *R.* sp. Davis 3056; *trnL* intron + *trnL-trnF* spacer), which most similar to accessions of *Tarenna* and lacks the characteristic mutations of *Rutidea* separating this genus and its putative sister genus *Nichallea* (represented as singleton) from all other Pavetteae accessions.

One accession labelled as *Canthium gynochthodes* (HG937663; *trnL* intron + *trnL-trnF*; Vanguerieae) is placed outside the subtrees including other accession of *Canthium* and placed within the *Psydrax* subtree (same tribe). *Psydrax* is sufficiently different in the covered fragment and has a high topological coherence. The accession is likely mislabelled or the species placed in the wrong genus.

Further candidates within the Vanguerieae for mislabelled, misplaced accessions are:

- KT218914 (*Fadogia* sp. Van Caekenberghe 349) → *Vangueria*;
- AJ874942 (*Fadogia agrestis*), affinity of the *trnL-trnF* fragment lies with *Vangueria* or closely related genera;
- FN386337 (*Peponidium* sp. G. Gautier 4632), → *Rhytigynia*; far the most accessions of *Rhytigynia* (50 out of 52) are placed in a unambiguously supported (BS_{ML} = 99, prominent root branch) clade including *Fadogia* and *Fadogiella*, and readily distinct from the genera included in the clade with other *Peponidium* accessions;
- EU821641 (*Psydrax schimperiana*), → *Keetia*, the other accession of the same species shows all characteristics of a *Pysdrax* sequences and is placed in the according clade of the Vanguerieae subtree. *Keetia* and *Psydrax* are sufficiently distinct in the sequenced fragment;
- KU853235 (*Psydrax sp*. Q2U44), → *Keetia* (see above)
- AJ620173 (*Rytigynia fuscosetulosa*) and AJ874959 (*Rytigynia induta*), → *Vangueria*

Table S1-3. Coherence of tribes represented by more than a single species in the trnTLF intron guide tree (meta-information as provided in gene bank flatfiles, following the current NCBI taxonomy tree; actual association may be different)

Tribe	Inclusive subtree	N(accessions)	N(genera)	Genera placed in according subtrees	Singletons	Separated by branches with BS ≥ 50
Alberteae	No[a]	25	5	5	0	0
Aulacocalyceae	No[b]	6	2	1	0	0
Coffeeae	No[c]	83	7	4	0	0
Condamineeae	Yes[d]	44	8(+1)	4	4	0
Gardenieae	No[e]	213	76	29	18	2
Ixoreae	Yes[f]	212(+17)	3(+3)	2(+2)	0(+1)	0
Mussaendeae	Yes	18	5	2	3	0
Octotropideae	No[g]	21	9	3	2	0
Pavetteae	Yes[h]	119	10	5	1	0[i]
Sabiceeae	Yes	61	4	2	1	0
Sipaneeea	No[j]	17	5	2	2	1
Vanguerieae	Yes	374	27	7	6	3

[a] *Tennantia* deeply nested in the Pavetteae subtree, *Airosperma* resolved as part of a long-branched subtree including *Boholia* forming the sister to a large clade including unclassified taxa (*Augusta, Wendlandia*), Alberteae, Aulacocalyceae, Coffeae, …

[b] The two genera are placed far apart from each other, with the *Aulacocalyx* accessions placed in poorly supported part of the tree.

[c] The subtree includes also *Bertiera* (only representative of Bertiereae), but not *Petitocodon* (cf. footnote [b], Table S1-1)

[d] The subtree includes also *Pentagonia* (classified as incertae sedis)

[e] The Gardenieae form a large grade, with other tribes and genera embedded within.

[f] The clade comprising all three Ixoroideae genera also includes also the unclassified genera *Aleisanthia, Aleisanthiopsis*, and *Greeniopsis.*

[g] All genera classified as Octotropideae are collected in the same subtree of the larger Gardenieae subtree, but mixed with genera not classified as Octotropideae.

[h] The Pavetteae subtree includes a few accessions of genera classified as Gardenieae.

[i] The large, well-sampled genera *Coptosperma, Pavetta* and *Tarenna* are not resolved but part of a large grade including smaller genera

[j] *Platycarpum* is placed in a sisterclade to the Sipaneeae clade. The comprehensive subtree includes also *Posoqueria* (only representative of Posoquerieae) and *Henriquezia* (not classified).

References

1. Göker M, García-Blázquez G, Voglmayr H, Tellería MT, Martín MP: **Molecular taxonomy of phytopathogenic fungi: a case study in *Peronospora*.** *PLoS ONE* 2009, **4**:e6319.
2. Katoh K, Standley DM: **MAFFT multiple sequence alignment software version 7: improvements in performance and usability**. *Mol Biol Evol* 2013, **30**:772–780.
3. Maddison WP, Maddison DR: **Mesquite: a modular system for evolutionary analysis**. In., http://mesquiteproject.org, 2.75 edn; 2011.
4. Stamatakis A: **RAxML version 8: a tool for phylogenetic analysis and post-analysis of large phylogenies**. *Bioinformatics* 2014, **30**:1312–1313.
5. Pattengale ND, Masoud A, Bininda-Emonds ORP, Moret BME, Stamatakis A: **How many bootstrap replicates are necessary?** In: *RECOMB 2009.* Edited by Batzoglou S, vol. 5541. Berlin, Heidelberg: Springer-Verlag; 2009: 184–200.
6. Hershkovitz MA, Zimmer EA, Hahn WJ: **Ribosomal DNA sequences and angiosperm systematics.** In: *Molecular Systematics and Plant Evolution.* Edited by Hollingsworth PM, Bateman RM, Gornall RJ. London: Taylor & Francis; 1999: 268-326.
7. Hershkovitz MA, Zimmer EA: **Conservation patterns in angiosperm rDNA ITS2 sequences**. *Nucl Acids Res* 1996, **24**(15):2857-2867.
8. Grimm GW: **Tracing the mode and speed of intrageneric evolution - a case study of genus *Acer* L. and *Fagus* L.** *D.Sc.* Tübingen: Eberhard-Karls University; 2003.

Fig. S1-2. Maximum likelihood (ML) guide tree based on partial 5′ ETS data of Ixoroideae

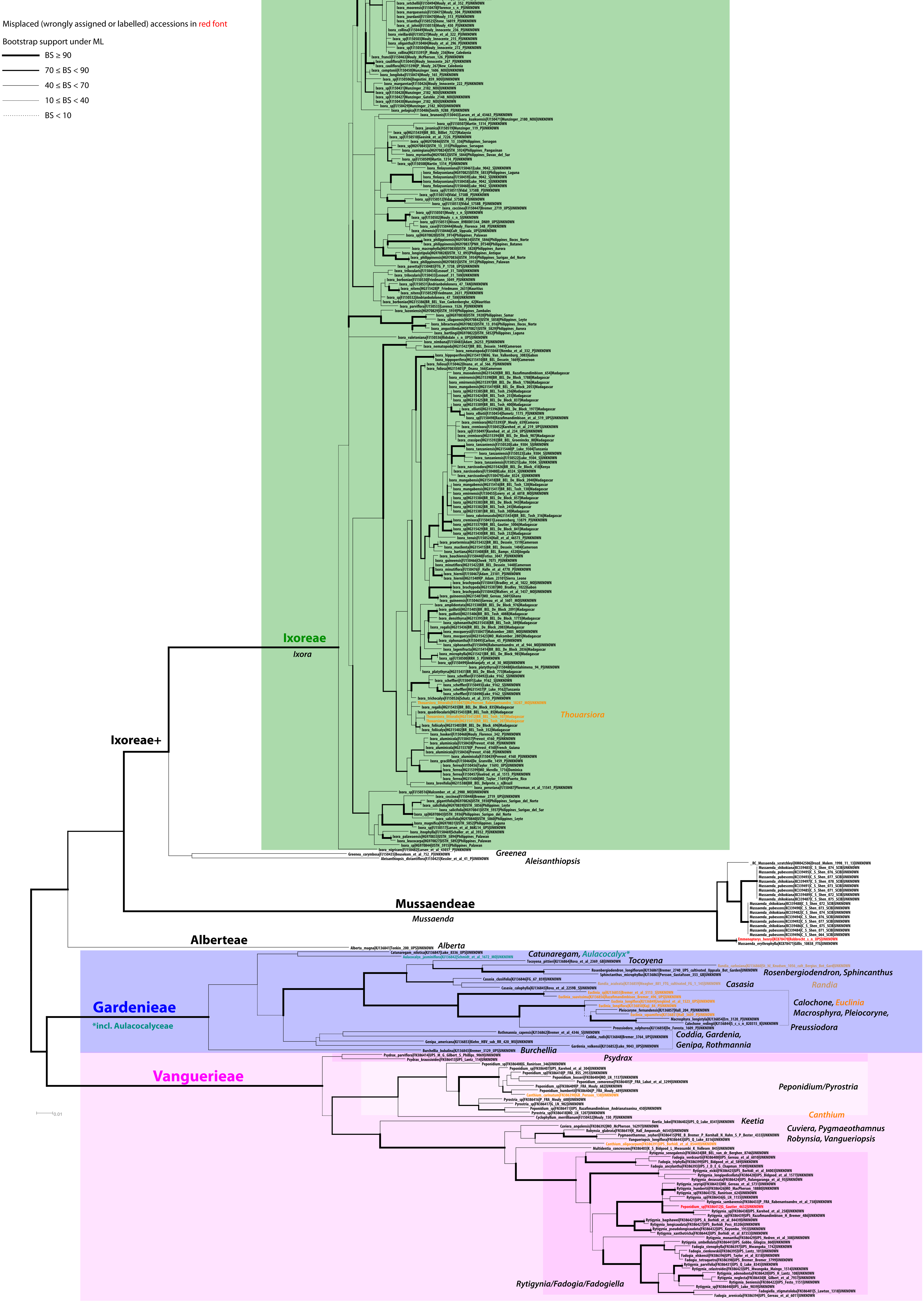

Fig. S1-3. Maximum likelihood (ML) guide tree based on ITS data (non-alignable, length-polymorphic portions excluded) of Ixoroideae

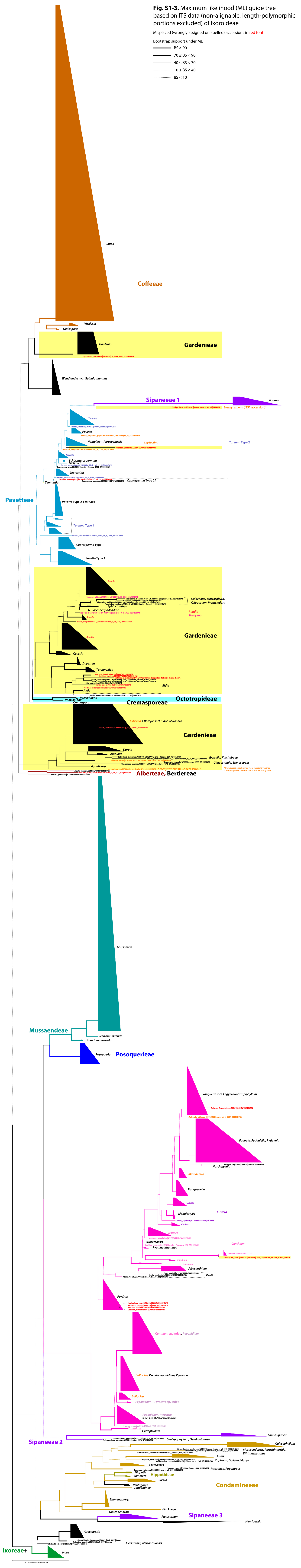

Fig. S1-4. Maximum likelihood (ML) guide tree based on *rps16* intron data of Ixoroideae

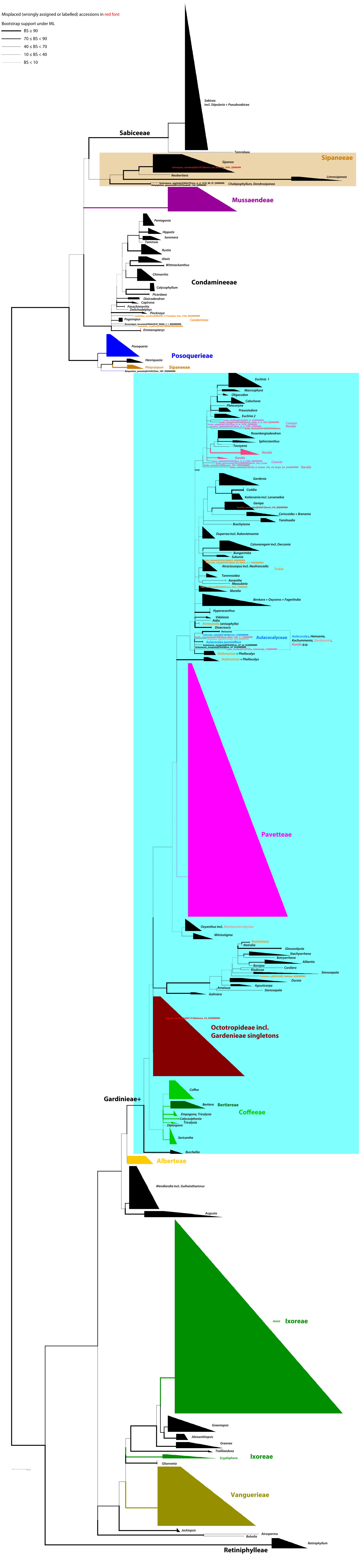

Fig. S1-5. Maximum likelihood (ML) guide tree based on concatenated data from the trnT-F region of Ixoroideae (*trnT-trnL* and t*rnL-trnF* spacers, and complete *trnL* gene)

Misplaced (wrongly assigned or labelled) accessions in red font

Bootstrap support under ML

- BS ≥ 90
- 70 ≤ BS < 90
- 40 ≤ BS < 70
- 10 ≤ BS < 40
- BS < 10

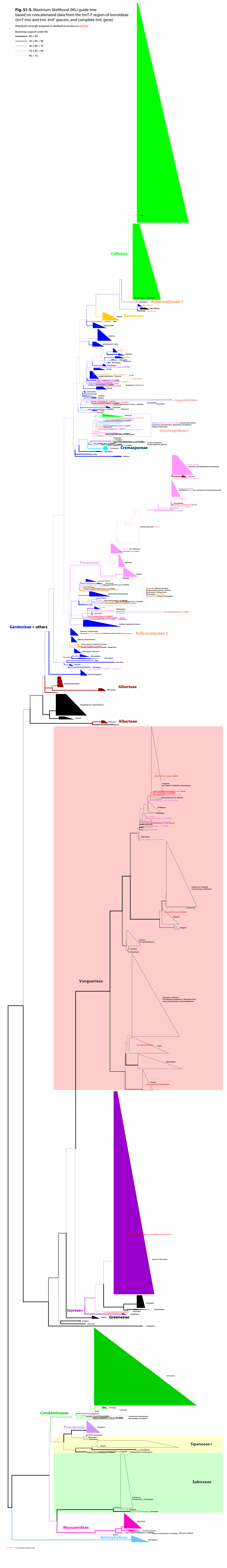

Supplement: Supplementary file 1 — Details relating to the curation and analysis of the data harvested from gene banks to confirm selection of best-suited outgroups (PDF 1128 kb) [file 12862_2017_974_MOESM1_ESM.pdf]
